# Global Regulator MorA Affects Virulence-Associated Protease Secretion in *Pseudomonas aeruginosa* PAO1

**DOI:** 10.1371/journal.pone.0123805

**Published:** 2015-04-20

**Authors:** Ayshwarya Ravichandran, Malarmathy Ramachandran, Tanujaa Suriyanarayanan, Chui Ching Wong, Sanjay Swarup

**Affiliations:** 1 Metabolites Biology Lab, Department of Biological Sciences, National University of Singapore, Singapore 117543; 2 Mechanobiology Institute, National University of Singapore, 5A Engineering Drive 1, Singapore 117411; 3 NUS Environmental Research Institute (NERI), National University of Singapore, 5A Engineering Drive 1, Singapore 117411; 4 Singapore Centre on Environmental Life Sciences Engineering (SCELSE), Nanyang Technological University 60 Nanyang Drive, SBS-01N-27 Singapore 637551

## Abstract

Bacterial invasion plays a critical role in the establishment of *Pseudomonas aeruginosa *infection and is aided by two major virulence factors – surface appendages and secreted proteases. The second messenger cyclic diguanylate (c-di-GMP) is known to affect bacterial attachment to surfaces, biofilm formation and related virulence phenomena. Here we report that MorA, a global regulator with GGDEF and EAL domains that was previously reported to affect virulence factors, negatively regulates protease secretion via the type II secretion system (T2SS) in *P*. *aeruginosa* PAO1. Infection assays with mutant strains carrying gene deletion and domain mutants show that host cell invasion is dependent on the active domain function of MorA. Further investigations suggest that the MorA-mediated c-di-GMP signaling affects protease secretion largely at a post-translational level. We thus report c-di-GMP second messenger system as a novel regulator of T2SS function in *P*. *aeruginosa*. Given that T2SS is a central and constitutive pump, and the secreted proteases are involved in interactions with the microbial surroundings, our data broadens the significance of c-di-GMP signaling in *P*. *aeruginosa* pathogenesis and ecological fitness.

## Introduction

*Pseudomonas aeruginosa* is a highly versatile opportunistic pathogen for humans and is a major cause of nosocomial infections in immunocompromised patients such as those suffering from cystic fibrosis, pneumonia and skin-burn. It mainly colonizes the respiratory tract, urinary tract, skin and surgical implants leading to high mortality rates in many cases [1]. Clinical isolates of *P*. *aeruginosa* are invasive or cytotoxic, with some cytotoxic strains also being inherently capable of invasion to some extent [2, 3]. The three classical stages of infection are (i) bacterial attachment to host cell and its colonization, (ii) local infection by tissue penetration and internalization, followed by (iii) dissemination via bloodstream [4]. The initial stages of tissue penetration and cellular invasion are especially critical for survival of bacteria and establishment of infection [5]. The non-mucoid *P*. *aeruginosa* PAO1 strain is known to effectively invade host cells and its efficiency of invasion is independent of lipopolysaccharide production or cytotoxicity [6]. While tissue penetration requires cleavage of extracellular matrix proteins and tight junctions, cellular invasion happens mostly through receptor-mediated response by the host [7]. Pathogenic bacteria accomplish these by releasing an arsenal of diffusible factors into the surrounding environment and delivering effector proteins directly into the host cytosol, through virulence-associated secretion systems on the surface. Extracellular proteins including toxins, proteases, lipases and lysins, which get secreted into the culture supernatant, are collectively referred to as the ‘secretome’. Given the flexible lifestyles and adaptability of *P*. *aeruginosa*, it is not surprising that it possesses five out of the six secretion machineries described to date in Gram-negative pathogens [8]. However their copy numbers and functional organization vary depending on the strain and its environment. Hence, it is a good model to study secretion processes and their control mechanisms.

The general secretory pathways in *P*. *aeruginosa* generally employ a two- step process to secrete proteins into the extracellular medium via a transient periplasmic intermediate. The first step of inner membrane translocation is carried out by the Sec and Tat (co-factor bound proteins) systems [9, 10]. The second step, subsequent transport beyond the periplasm via the type II secretion system (T2SS) is a well-known mechanism [11]. Since the substrates of T2SS include both virulent factors and degradative enzymes, it plays a central role in pathogenesis and adaptation [12– 15]. The T2S multi-protein nanomachine, also termed ‘secreton’, spans both the inner and outer membranes across the periplasm and is highly conserved among Gram-negative bacteria [16, 17]. It is a complex, typically composed of 12 proteins that make-up four subassemblies namely the pseudopilus, the outer-membrane complex, the inner-membrane platform and the secretion ATPase [18, 19]. However, the molecular model of the secretion mechanism is yet to be established [20]. There are four potential T2SS systems in *P*. *aeruginosa* [21–23], of which the Xcp system is the most studied [24]. In *P*. *aeruginosa*, the number of assembled secretion machineries is estimated at 50–100 complexes per cell [25] that are polar-localized [26]. Powered by ATPase activity at the inner membrane, the pseudopilus acts as a piston to export proteins from the periplasm through the outer-membrane pore [18, 27]. Exoproteins that use the T2SS are characterized by the presence of a signal peptide at their N-terminus, which gets proteolysed at the periplasm before getting secreted [28–30].

*P*. *aeruginosa* employs multiple regulatory mechanisms such as two-component systems, transcriptional regulators, sigma factors and small molecule signaling for the coordinate control of its virulence determinants in response to a wide range of environmental cues [31]. These can act at transcriptional, translational or post-translations levels. One such mechanism is the cell-cell communication system called quorum sensing [32], which regulates expression of a considerable number of genes in response to a critical concentration of signal molecules representative of the density of bacterial population [33, 34]. Expression of genes encoding T2SS machinery (*xcp*) [35] and substrate proteins exported through it have been reported to be under of the control of two QS systems namely *lasRI* and *rhlRI* [36, 37]. Correspondingly, the extracellular levels of several secreted proteins including T2SS substrates are governed by these QS systems as well [38]. The regulation via QS is complex and is controlled by Vfr, a homologue of *Escherichia coli* cyclic AMP receptor protein (CRP) [39].

Likewise, the signal transduction pathway mediated by second messenger cyclic diguanylate (c-di-GMP) has well-established impact on multifarious virulence mechanisms in a wide variety of bacteria [40–42] including surface transport systems such as flagella biogenesis [43], adhesin production [44, 45] and type III secretion system (T3SS) [46] in *P*. *aeruginosa*. Evidence exists for varied modes of regulation by c-di-GMP namely transcriptional, post-transcriptional and translational [47–51]. Some recent studies have also established its involvement in regulating secretion machineries. C-di-GMP levels have been demonstrated to be critical for periplasmic processing of adhesin LapA in *P*. *fluorescens* [52], switching bacterial lifestyles by modulating T3SS and T6SS [53] as well as the Type I secretion machinery of a phytopathogen [54] and linked to type VI secretion system in a fish pathogen [55].

Previously, we have described a membrane-localized motility regulator, MorA, which possesses domains that are involved in the turnover of c-di-GMP, namely diguanyate cyclase (GGDEF motif) and phosphodiesterase (EAL motif). We have shown that MorA controls the timing of flagellar development by restricting flagellin (*fliC*) expression and hence affects motility, chemotaxis, and biofilm formation in *P*. *putida* PNL-MK25 [56]. In *P*. *aeruginosa* PAO1, the absence of MorA (PA4601) led to a reduction in biofilm formation [56]. Further investigation by expression profiling revealed the possibility that many surface-associated phenomena, particularly secretion-related genes might be under the control of MorA in *P*. *aeruginosa* [57]. MorA has also been linked to fimbriae formation in a clinical strain [58]. A recent report has confirmed that MorA is predominantly a diguanylate cyclase (DGC) with some phosphodiesterase (PDE) activity *in vitro* [59]. It is believed that the PDE domain could have a regulatory role *in vivo* likely through dimerization. Thus, we explored whether MorA plays a significant role in regulating *P*. *aeruginosa* secretome. In this study, we have investigated the effect of MorA on protein secretion in planktonic *P*. *aeruginosa* PAO1 cultures and show evidence suggesting that MorA negatively controls T2SS-mediated protease secretion, which in turn impacts infection efficiency of *P*. *aeruginosa*. While the GGDEF domain of MorA is predominantly involved in this regulation, interestingly, the EAL domain also seems to have a similar but lesser effect and both likely occur at the post-translational level.

## Materials and Methods

### Bacterial strains, plasmids and growth conditions

The bacterial strains and plasmids used in this study are described in Table 1. *P*. *aeruginosa* PAO1 [60] cultures were grown aerobically at 37°C in Luria-Bertani (LB) medium (1.0 Tryptone, 0.5% Yeast Extract, 1.0% NaCl; pH 7.0) unless stated otherwise.

**Table 1:** Bacterial strains and plasmids used in this study.

Strain/ Plasmid	Characteristics		Source/Reference
***P*. *aeruginosa* strains**			
PAO1 WT		Wild-type *P*. *aeruginosa* strain	[60]
*morA* KO		PAO1 with insertional mutation in *morA* (*morAPa*::*aacC1*); Gm^r^	[56]
Δ*morA*		Markerless deletion mutant of *morA* in PAO1	This study
Δ*morA*-pU		Δ*morA* complemented with full length *morA* (pUPMR)	This study
Δ*morA*-pUG*		Δ*morA* complemented with full length *morA* containing GGDEF domain mutation (pUE1060K)	This study
Δ*morA*-pUE*		Δ*morA* complemented with full length *morA* containing EAL domain mutation (pUE1189K)	This study
*fliC* KO		PAO1 with insertional mutation in *fliC* (fliC:: Gm^r^)	[67]
*xcpQ*		PAO1 mutant strain PW6222 (xcpQ-C07:: ISphoA/hah)	PA two-allele library [74]
***E*. *coli* strains**			
BL21		F-, *ompT*, *hsd*S(r^-^_B_, m^-^_B_), *gal*, *dcm*; host for protein expression	Laboratory collection
DH5α		F– Φ80*lac*ZΔM15 Δ(*lac*ZYA-*arg*F) U169 *rec*A1 *end*A1 *hsd*R17 (rK–, mK+) *pho*A *sup*E44 λ– *thi*-1 *gyr*A96 *rel*A1; host for pK18*mobsacB* and pK-*morA*flank	Laboratory collection
**Plasmids**			
pUPMR		Full-length *morA*_*Pa*_ gene with native promoter cloned into pUCP19; Amp^r^	[56]
pRK2013		Vector that aids mobilization of plasmid in triparental conjugation, kan^r^ Gm^r^	[62]
pK18*mobsacB*		Allelic exchange suicide plasmid, sucrose-sensitive, kan^r^ Gm^r^	[61]
pK-*morA*flank		pK18*mobsacB* with fusion product containing 5’ upstream and 3’ downstream regions of *morA*, kan^r^ Gm^r^	This study
pUE1060K		pUPMR with E1060K mutation; Cb^r^	This study
pUE1189K		pUPMR with E1189K mutation; Cb^r^	This study
pETM		Modified pET32 (Novagen) expression vector lacking Trx and S tags	Laboratory collection
pETM-LasB		pETM carrying partial *lasB* (A_198_-L_498_)	This study

A markerless *morA* null mutant was generated by allelic replacement using pk18mobsacB [61]. The 5’ and 3’ flanking chromosomal regions of *morA* gene were amplified using primers with overlapping XbaI restriction site and ligated into a single fragment, which was in-frame to exclude the ORF. The resulting fragment was cloned into the pk18*mobsacB* resulting in a suicide construct (pK-MorAflank) and was transformed into *E*. *coli*. Mobilization into *P*. *aeruginosa* PAO1 WT was performed by triparental conjugation in 1x M9 minimal medium using pRK 2013 [62] as the helper strain. Selection for single cross-over recombination was done on LB supplemented with Gentamycin. These primary Gm^R^ transconjugants were verified by PCR and again grown in the absence of Gm to allow double cross-over recombinations. Upon counter selection on medium containing 5–10% sucrose, the colonies were further tested for loss of Gm resistance. Mutants that were both sucrose resistant and Gm sensitive were screened by PCR using primers that flank the *morA* gene. The products were sequenced to confirm the markerless deletion of *morA*, denoted as *ΔmorA* in this study.

For the generation of point mutants of *morA* domains (E1060K and E1189K), primers introducing the site-directed mutations were used to generate partial *morA* fragments, which were subsequently fused via PCR amplification using forward and reverse primers for full-length *morA* gene. Primer and gene positions of the partial fragments can be found in S1 Table. Amplicons carrying these mutations were cloned into pUPMR [56] at the AscI and NcoI restriction sites. These pUPMR plasmids carrying point mutations were subsequently introduced into *ΔmorA*.

### Extraction of cell-associated protein fraction

Bacterial pellets from cultures used for extra-cellular protein extraction were washed once and resuspended in a maximum volume of 3 ml extraction buffer (50 mM Tris, 1 mM EDTA, 20 mM DTT) with Complete, Mini Protease Inhibitor Cocktail (Roche). Homogenization was carried out in a cell disrupter Micro Smash MS-100 (Tomy Seiko Co. Ltd., Japan) with 0.1 mm glass beads in screw cap tubes at 20 sec pulses of 4,000 rpm repeated about 8–10 times until the pellet was completely disrupted. The tubes were then centrifuged at maximum speed on tabletop centrifuge for 10 minutes, supernatant saved. This is referred to as the ‘cellular fraction’ in this article. Equal volumes of protein were loaded for SDS-PAGE.

### Secretome analysis

For analysis of secreted protein levels, the different strains were cultured at 37°C in equal volume Luria-Bertani (LB) medium till they reached the same optical density (OD_600_). For late-log and mid-log phase cultures, the growth time was about 13 hrs and 9 hrs respectively. A physical cell count was also performed using hemocytometer to verify the OD_600_ measurement. Trichloroacetic acid (TCA) precipitation method was used to extract extracellular proteins (ECP) from culture supernatants as described previously [63]. Briefly, TCA was added to the cell-free supernatants to a final concentration of 10% w/v and incubated at 4°C for 2–16 hours. The precipitated protein was recovered by centrifuging at 20,000 x g for 5 minutes at 4°C and the pellet washed thrice with ice-cold acetone to remove salts. Upon air-drying, all the pellets were dissolved in equal volume of a denaturing buffer containing 40 mM Tris, 40 mM Dithiothreitol (DTT) and 2% SDS and loaded using a buffer devoid of SDS for PAGE analysis.

Equal volumes of ECP samples were loaded for SDS-PAGE. Further, to account for inaccuracies and ensure protein loading from equal number of cells, minor volume adjustments (5–10%) were made based on the intensity of RNA polymerase band (immunoblot) from the respective cellular fractions. Densitometry of relative levels of protein bands was performed using the image analysis tool ImageJ 1.43 (http://rsbweb.nih.gov/ij/). Bands were selected for quantification based on contrast difference with respect to the background. Intensity values obtained as area under curve were converted to relative proportions. Gel strips were then sent to Protein and Proteomics Centre, Department of Biological Sciences, National University of Singapore for identification of individual proteins by using 4800 MALDI TOF/TO Analyzer (Applied Biosystems). Peptides were matched using Mascot search against *P*. *aeruginosa* PAO1 database of NCBI.

### Elastolytic activity assay

Cell-free supernatants were concentrated 200 times using centrifugal filters (Ultracel-10k, Millipore) and total ECP concentration estimated by Bradford method (Bio-Rad). Elastase activity assay was performed using a modification of a method described previously [64]. For each sample, 5μg protein was added to 20 mg Elastin-Congo red (Sigma) suspended in 1ml of reaction buffer (25 mM Tris pH 7.8, 0.15 mM NaCl, 10 mM CaCl_2_) and incubated with slow rotation at 37°C for 6 hrs. The assay tubes were then centrifuged at low speed to remove insoluble material and absorbance of the supernatants measured at 495nm. *Pseudomonas aeruginosa* elastase (Elastin Products Company, Inc., USA) was used as standard to calculate activity (S1 Fig).

### Membrane protein preparation

The membrane proteins were prepared as described previously [65] with some optimization. Overnight *P*. *aeruginosa* culture in LB was pelleted down at 5000 g at 4°C for 10 minutes. The cell pellet was washed once with 50mM sodium phosphate buffer (pH 8) and resuspended in the same buffer (8ml per 50ml culture) containing Complete, Mini Protease Inhibitor Cocktail (Roche). Homogenization was optimized to 4 min (12 x 20 sec pulse) of sonication at maximum intensity to ensure complete cell disruption. The homogenized suspension was centrifuged at 1,500 x g at 4°C for 10 minutes to remove cell debris. The clear supernatant was centrifuged again at 125,000 x g at 4°C for 30–45 minutes to separate the membrane fraction as pellet and the cytoplasmic and periplasmic fractions in the supernatant. The pellet was then dissolved in the extraction buffer with protease inhibitor cocktail (Roche) and stored in -80°C until further use.

### LasB antibody production

Recombinant LasB corresponding to A_198_-L_498_, which lacks the signal peptides, was expressed in *E*. *coli* BL21 carrying a modified version of the expression plasmid pET32 (Novagen), pETM. Primer details can be found in S1 Table. A mixture of this recombinant partial protein and secreted LasB extracted from culture supernatants were used for polyclonal antibody production by LAMPIRE Biological Labs. Inc., USA.

### Immunoblot analyses

The XcpP, XcpY and XcpZ rabbit antibodies were generous gifts from Dr. Gerard Michel, CNRS, France [65, 66]. Mouse monoclonal antibody against alpha subunit of *E*. *coli* RNA polymerase was purchased from Neoclone Biotechnology, WI, USA and used as control. LasB antibody was produced as part of this study. All secondary antibodies were anti-rabbit/mouse IgG conjugated with alkaline phosphatase (Sigma). Immobilon Western chemiluminescent AP substrate (Millipore) was used as substrate.

### Transcriptional analysis by qRT-PCR

Total RNA were extracted from bacterial pellets using TRIzol Reagent (Invitrogen Corp., USA) according to manufacturer’s instructions with some modifications. Bacterial cell pellets were mixed by vortexing in TRIzol and heated at 50°C for 10 min prior to RNA extraction to lyze the cells. RNA samples were prepared from early-, mid- and late-logarithmic phases. cDNA was prepared using the Superscript II First-Strand Synthesis System (Invitrogen) as per manufacturer’s instructions. RNA concentrations were determined using a Nanodrop (Nanodrop Technologies, USA). Real-time quantitative PCR was performed on an ABI Prism 7700 thermal cycler using SYBR Green PCR Master Mix (Applied Biosystems) as per manufacturer’s instructions. Gene-specific primers designed using Primer Express (Applied Biosystems) were used (S1 Table) and *RpsL*, a gene that encodes a ribosomal protein, was used as the endogenous control. The comparative C_T_ method (ΔΔC_T_) for relative quantification of gene expression was used for calculating fold change. All amplifications were done with three independent growth experiments (biological replicates) and two RNA samples (technical replicates) from each of them.

### Cell culture conditions and invasion assay

Lung fibroblast line MRC-5 (American Type Culture Collection CCL-171) monolayer was grown in Eagle's Minimum Essential Medium containing 10% fetal bovine serum (Hyclone) and penicillin (100 IU/mL)- streptomycin (100 μg/mL) at 37°C. For infection with *P*. *aeruginosa* PAO1 strains, bacterial cells at log phase (OD_600_ 0.8–1.2) were collected, washed and resuspended in PBS. Immediately, a cell count was performed to determine the volume of bacterial suspension to infect the host cell culture. After allowing infection for 1 hr, unattached swimming bacteria were washed off using PBS.

For invasion assay, antibiotics of higher concentration, namely 250 IU/mL penicillin- 250 μg/mL streptomycin was used for efficient killing of bacteria attached to the surface of the host cells. After incubation at 37°C for 1 hr, the cells were washed twice with PBS to remove antibiotics, lysed with 100 μl of 1% TritonX-100 and the contents homogenized by pipetting. This suspension containing the internalized bacteria was serially diluted and plated on LB agar plates. After ~24 hr incubation in 37°C, viable colony count was performed. Efficiency of invasion was calculated as the difference between the number of bacteria used for infection and the number internalized divided by number of bacteria used for infection. Infection time and multiplicity of infection (MOI) were standardized to achieve optimal conditions that resulted in significant difference in invasion. However, the percentage range of invasion efficiency between the two experimental sets varied, likely due to difference in their scale. Infection was performed in 24-well plates for the set with *fliC* KO control [67] and in 12-well plates for the set with different infection conditions. Hence, the absolute numbers of host and bacterial cells were used based on OD values, which could have led to the variability.

## Results

### MorA affects levels of extracellular proteins in *P*. *aeruginosa* PAO1

**Fig 1:**
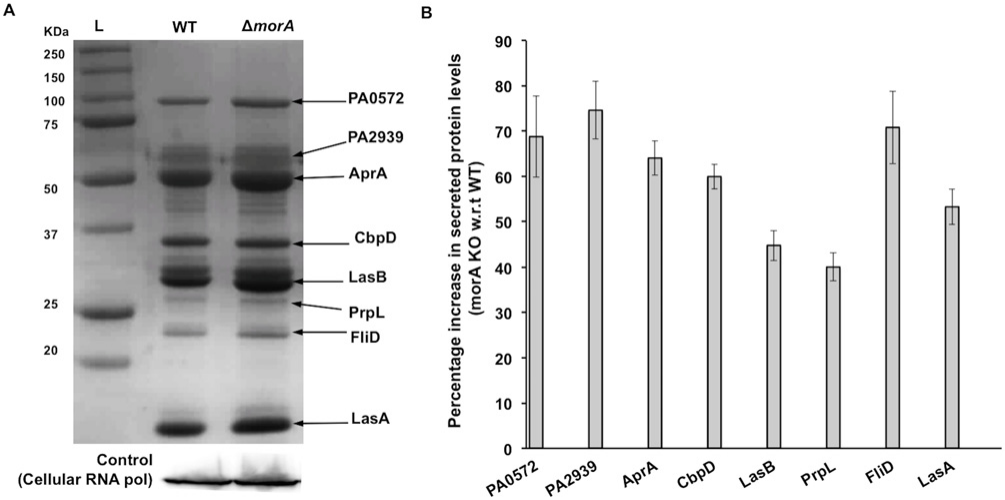
Levels of secreted proteins are affected by MorA in *P*. *aeruginosa*. (A) Top panel—Profiles of total extracellular protein (ECP) from *P*. *aeruginosa* PAO1 WT and *morA* KO culture supernatants. Samples were loaded based on protein secreted from equal number of cells. Protein bands were identified by MALDI-ToF-ToF. Bottom panel—Immunoblot of RNA polymerase (loading control) from cellular fractions of respective cultures. L-Protein ladder (Bio-Rad). (B) Percentage increase in levels of secreted proteins in *morA* KO compared to WT. Error bars represent mean +SE (n = 3). Student’s t-test, p-value<0.05.

**Fig 2:**
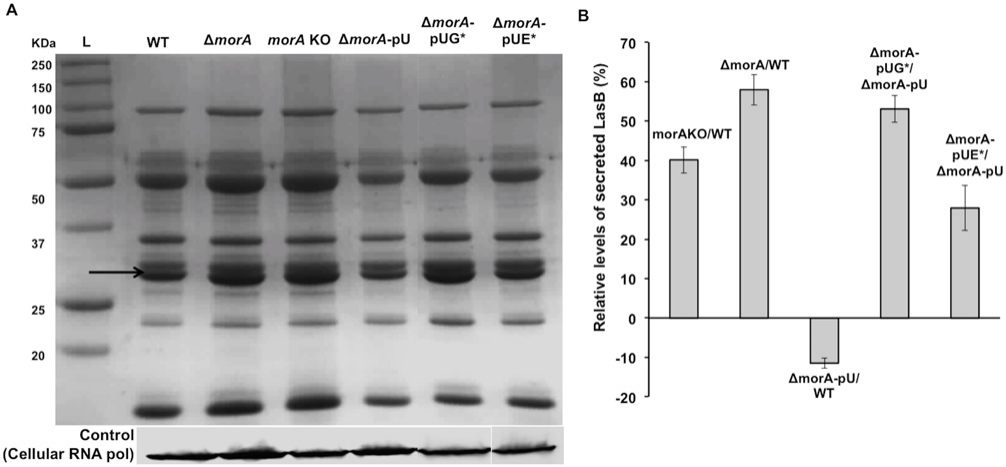
Effect on protein secretion by MorA in *P*. *aeruginosa* is c-di-GMP signaling dependent. **(**A) Top panel—Total extracellular protein (ECP) from culture supernatants of *P*. *aeruginosa* strains loaded based on protein secreted from equal number of cells. Black arrow indicates the position of elastase (LasB) band. Bottom panel—Immunoblot of RNA polymerase (loading control) on cellular fractions of respective cultures in top panel. L-Ladder (Bio-Rad). (B) Percentage increase in levels of secreted LasB based on protein band quantification by densitometry. Band intensity values of MorA insertion (*morA* KO) and deletion (Δ*morA*) mutants are compared with wildtype (WT) while those of strains expressing MorA with mutations in GGDEF and EAL motifs (represented as Δ*morA*–pUG* and Δ*morA*–pUE* respectively) are compared with complementation strain (Δ*morA*-pU) containing full length *morA* expressed in Δ*morA* background. Error bars represent mean +SE (n = 5). Student’s t-test, p-value<0.05.

Based on our previous findings from gene expression profiling of MorA mutant [57] and other reports showing evidence that c-di-GMP regulating proteins control bacterial secretion systems [45, 46, 52–55], we tested the effect of MorA on the *P*. *aeruginosa* secretome. Profiles of extracellular proteins of *P*. *aeruginosa* PAO1 WT and *morA* KO were compared. RNA polymerase α-subunit levels in cellular fractions from the same samples were used as controls for biomass. Strains lacking MorA had higher overall levels of extracellular proteins than WT in planktonic culture supernatants (Fig 1A). In order to quantitate the levels of extracellular proteins, densitometry analysis of individual bands from the protein profiles was performed. As shown in Fig 1B, there was at least 50% increase in secretion due to *morA* mutation in six out of eight proteins observed. Decrease in levels of extracellular proteins upon *morA* complementation (Fig 2) further validated that changes in their levels were due to MorA expression.

Identity of the extracellular protein bands that showed differences in intensities between strains was established by MALDI-ToF-ToF. Spectra and peptide information can be found in S2 Fig and S2 Table. Five out of eight identified proteins were proteases. All the five proteases and the chitin binding protein are known to be secreted by the Type II secretion system [24, 68–70]. We, therefore, refer these proteins collectively as secreted proteins or secretome. Elastase LasB, the major protease secreted via T2SS showed nearly 40% increase in *morA* KO than WT.

### MorA controls T2SS secretome levels via its c-di-GMP signaling domains

In order to further ascertain the role of MorA-mediated c-di-GMP signaling in the differential secretion phenotype, secretome profiles of strains carrying MorA deletion strain (Δ*morA*) or those expressing MorA with mutations in GGDEF or EAL motifs were analyzed. The band intensity of elastase, the representative protease, was quantified for comparison between the strains.

Both insertion (*morA* KO) and deletion (Δ*morA)* mutants revealed similar trends of increase in secreted elastase levels (Fig 2A and 2B). Further comparison of point mutants of GGDEF and EAL domains in the *morA* null background- Δ*morA*-pUG* and Δ*morA*-pUE* respectively, with full length *morA* complementation (Δ*morA*-pU) revealed that the GGDEF mutant had higher secreted protein levels than the EAL mutant. The percentage difference in LasB levels for G* with respect to complementation strain was comparable to that observed for *morA* deletion strain with respect to WT, respectively (Fig 2B). Interestingly, E* also showed a similar qualitative trend (increase in LasB levels) as that of G*, albeit of lesser magnitude and was comparable to that of *morA* insertion mutant with respect to WT. These results provide evidence that the effect of MorA on *P*. *aeruginosa* secretion is dependent on its enzymatic domains, thereby implicating a role of c-di-GMP signaling in controlling T2SS secretome levels.

### Extracellular elastase activity increases with the loss of MorA

**Fig 3:**
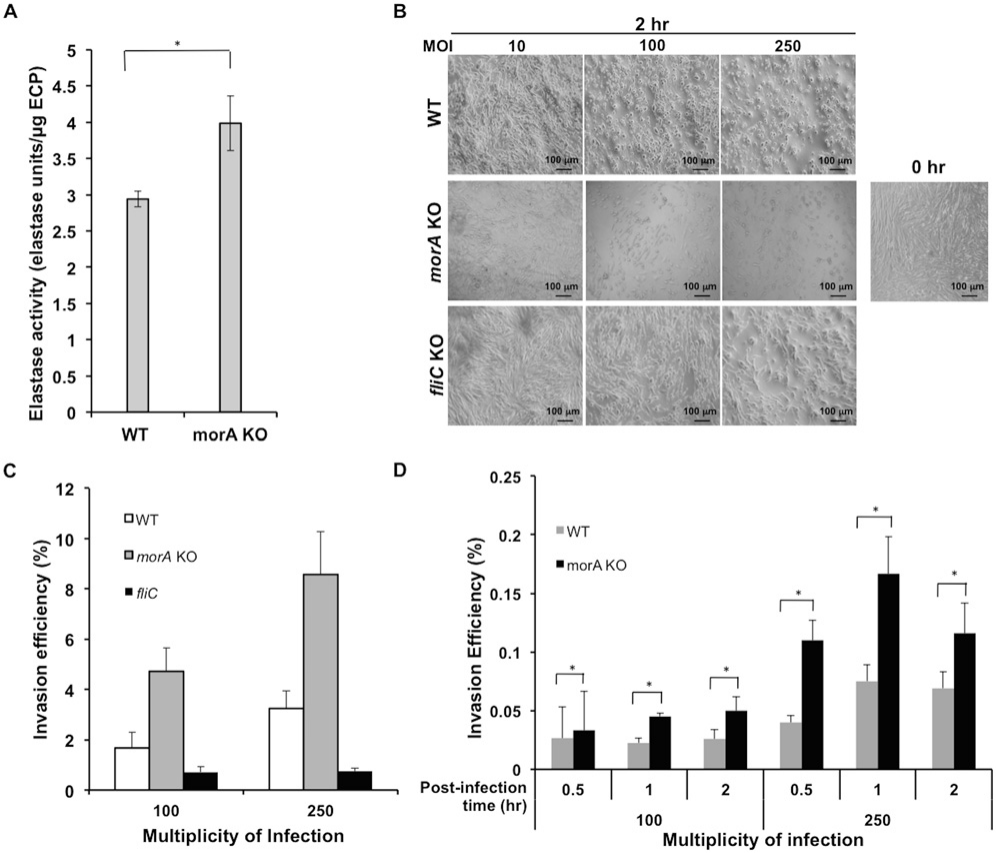
Elastase activity, host cell morphological changes and invasion efficiency corroborate with differential protein secretion. (A) Graph shows total active elastase per unit of total secreted extracellular proteins measured in *P*. *aeruginosa* PAO1 WT and *morA* KO. Activity was measured using elastin-congo red as substrate. Error bars represent mean +SE (n = 4). *Student’s t-test, p-value<0.05. ECP-extracellular protein. (B) Lung fibroblasts (MRC-5) infected with *P*. *aeruginosa* WT and mutant strains viewed by differential interference contrast microscopy. Images were captured 2 hours post-infection. MOI- Multiplicity of infection; 0 hr- No infection (control). (C) Graph shows difference in invasion efficiency of *P*. *aeruginosa* strains on lung fibroblasts (MRC-5) 2 hours post-infection. (D) Effect of MorA on invasion efficiency is consistent over a range of infection time and multiplicities of infection. Error bars represent mean +SE (n = 3). *Student’s t-test, p-value<0.05.

We tested whether the increased levels of extracellular proteases are also associated with increased protease activity. Since elastase is the key activator protease of many other extracellular proteases, its elastolytic activity in the extracellular protein fraction was chosen to be a representative of the cumulative effect of the secreted protease activity. The results showed a statistically significant increase of nearly 33% in the elastase activity in *morA* mutant compared to that in WT, which is consistent with the increase in elastase protein levels (Fig 3A).

### Invasion efficiency correlates with altered secretion due to MorA loss

Since protease activity plays a critical role in tissue penetration and colonization of *P*. *aeruginosa* in the host [71, 72], we tested the biological significance of altered protease secretion by testing the efficiency of bacterial invasion. Lung fibroblast cell line (MRC-5) was chosen as the model for infection by *P*. *aeruginosa* PAO1. Marked changes in host cell phenotype were evident between adherent cultures infected with WT and *morA* KO. Cell rounding and surface detachment as the proteases act on extracellular matrix (ECM) proteins were higher in mutant infected cultures than WT infected ones (Fig 3B). Since flagellar attachment to the host is also a crucial event preceding invasion, a mutant lacking the flagellar filament (*fliC* KO) was used as negative control for invasion, which exhibited poor attachment and highly limited invasion capacity.

The invasion efficiency of *P*. *aeruginosa morA* KO was at least two-fold higher compared to WT (Fig 3C). Invasion efficiencies at two different multiplicities of infection over time were calculated to verify the consistency of the effect of MorA signaling on the invasion phenotype (Fig 3D). The range of invasion efficiency due to MorA perturbation varied from 1.5–3 folds in most conditions and was statistically significant (p-value<0.05) (Fig 3C and 3D). Thus, the overall trend remained similar across tested conditions.

### Mode of regulation of *P*. *aeruginosa* T2SS function by MorA signaling

We hypothesized that the possible ways in which MorA might regulate the T2SS secretome levels could be by coordinately affecting: a) the RNA levels of protease genes, b) the cell-associated protease levels, c) the number of T2SS assemblies per cell, or d) the secretion efficiency of the machinery. Each of these hypotheses was addressed. RNA and protein levels of LasB elastase were quantified at different stages of planktonic growth since elastase secretion is known not to be uniform through the growth curve. Under our experimental conditions, secreted elastase was detectable only at the late-log to stationary transition phase. Earlier time points were included to analyze whether proteases accumulate in the cell before effective secretion takes place.

**Fig 4:**
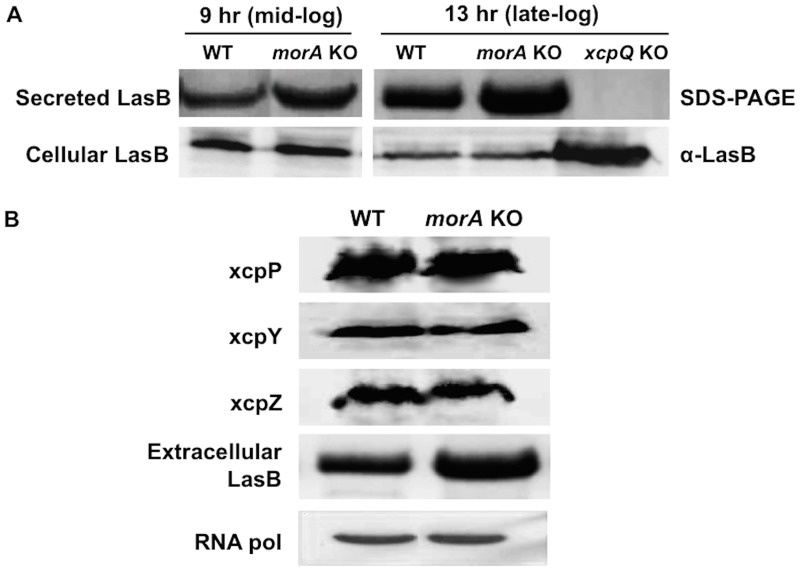
(A) MorA does not affect cellular levels of LasB. Top panel- Extracellular LasB from WT and *morA* KO culture supernatants at mid log and late log phases (SDS-PAGE). XcpQ mutant lacks functional T2SS and does not secrete any proteases; negative control. Bottom panel- LasB from cellular fractions of respective cultures immunoblotted using polyclonal antibody. Samples on both panels were loaded based on proteins from equal number of cells as described in methods. **(B) Levels of T2SS machinery proteins remain unaltered.** Immunoblots of the T2SS machinery component proteins from membrane fraction. Membrane proteins were loaded from equal number of bacterial cells. XcpY and XcpZ are inner membrane proteins while XcpP spans both the inner and outer membranes. Respective culture supernatants were loaded to compare secreted LasB levels. RNA pol- RNA polymerase from the cellular protein fraction was used as loading control.

Comparison of expression levels of genes encoding two key secreted proteins (LasB and CbpD) between *P*. *aeruginosa* PAO1 WT and *morA* KO revealed only a small increase (< 2-fold change of RNA levels; p-value<0.05) due to MorA loss (S3 Fig). It is to be noted that at LasB transcript levels were much lower in *morA* mutant than WT especially at late log phase beyond which secreted elastase was detected in the culture supernatants. At late log phase, the secreted protein levels (~60% increase; Fig 1B) and transcript levels (~30% increase) of CbpD due to *morA* mutation are not directly correlating. Further, unlike the extracellular protein levels, no significant change in the cell-associated levels of LasB was observed over time due to MorA loss (Fig 4A). Quantification of immunoblot using polyclonal LasB antibody on the cellular fraction confirmed that the difference due to *morA* mutation was less than 5%. A mutant defective in the T2SS outer membrane pore complex protein XcpQ, which is incapable of secretion [73, 74], had significant cellular accumulation, as expected (negative control) (Fig 4A). As there are small differences at RNA level too, a minor contribution of regulatory control at RNA level cannot be ruled out. However, as this does not proportionally reflect in protease production as seen in the cellular fractions, post-translational control seems to be the major control step for T2SS secretion by MorA.

We postulated that if MorA mutation was responsible for increased secretion by affecting the number of T2SS pumps, then it would be reflected in the corresponding level of T2SS component proteins. In such a case, the number of T2SS assemblies on the bacterial membrane per cell is expected to be higher than in WT. Hence, the levels of three key T2SS structural proteins, two on the cytoplasmic membrane- XcpY, XcpZ and one that spans across both the inner and outer membrane layers- XcpP, were compared in the membrane fraction. None of the proteins tested show significant change in their levels in membrane preparations of *P*. *aeruginosa* PAO1 WT and *morA* KO strains (Fig 4B). Densitometry analysis also confirmed similar levels of the secreton members (data not shown). Thus, increased expression of proteases or that of the secretion system cannot explain increased Type II secretome levels. These results, therefore, eliminate the above possibilities and strongly suggest that MorA-mediated c-di-GMP signaling likely acts at the level of secretion efficiency of T2SS.

## Discussion

The second messenger c-di-GMP signaling system is a well-known regulator of various cellular processes and virulence determinants in bacteria. Here, we report a novel mechanism for the control of protease secretion via T2SS by the c-di-GMP signaling domains of MorA, which operates largely at the post-translational level in *P*. *aeruginosa*. Notably, MorA loss results in a substantial increase of elastase (LasB), the major protease secreted by T2SS, in the extracellular fraction. Further, we have shown that the elastolytic activity is also correspondingly high in the extracellular fraction in *morA* mutants. Though the effect of MorA on elastase secretion is lesser compared to that due to loss of its transcriptional regulators [38], this might still have significant biological effects; the reason being that LasB is critical for the functional activation of several other secreted proteases such as LasA and aminopeptidase [75, 76]. Taken together, these results indicate that effect of c-di-GMP sensor regulator MorA on protease secretion is significant and biologically relevant during *P*. *aeruginosa* pathogenesis.

All the proteases that show differential secretion levels in this study have well-established roles in the penetration of host tissue and spread of disease [70–72]. LasB, a zinc metalloproteinase, cleaves a variety of host proteins at multiple sites in addition to elastin. Thus, it ruptures the respiratory epithelium by damaging tight-junctions and facilitating neutrophil recruitment. LasA is also a zinc metalloendopeptidase with low elastolytic activity, but a key player as it enhances the elastolytic activity of LasB. It is activated upon processing by LasB, alkaline protease or PrpL (protease IV) [72]. PrpL (PvdS-regulated endoprotease; lysyl class) is a serine protease that can digest casein, lactoferrin, transferrin, fibrinogen, plasminogen and decorin [77, 78]. CbpD (Chitin binding protein) is a non-staphylolytic protein, which is cleaved by elastase and is suggested to act as a adhesion-mediating colonizing factor of eukaryotic cells, although this remains to be proven [68]. The aminopeptidase (PA2939), which is enriched in the outer membrane vesicles (OMVs) of *P*. *aeruginosa* clinical isolates, may have a role in colonization of the lungs since OMVs are known to activate a significant pro-inflammatory response in lung epithelial cells [79]. Thus this finding highlights that c-di-GMP signaling by MorA affects a major virulence factor in this pathogen, which confers the unique invasive characteristic to it.

Interestingly, in this study, the site-directed mutants of MorA enzymatic domains (Δ*morA*-pUG* and Δ*morA*-pUE*) show the same qualitative trend for protease secretion as that of their roles in c-di-GMP turnover [59]. Hence, it is clear that the effect of MorA on T2SS is consistent with its enzymatic function i.e. its ability to regulate c-di-GMP levels. Infection assays show that the increased secretion phenotype due to MorA loss leads to increased invasion efficiency and thus has relevance at its function level. It is well established that flagellar attachment is vital for host cell invasion; thus one may argue that the changes in host cell morphology and invasion efficiency due to MorA loss could have resulted through modulation of the flagellar pathway. However, we have previously demonstrated that *morA* perturbation only led to impairment of biofilm and had no effect on flagellum number [56].

Investigation on underlying mechanisms and intermediary players linking MorA and T2SS has ruled out the secretion machinery components as well as its secreted substrates at the transcriptional and translational level. Based on our current understanding, this is the first report on post-translational level regulation of protease secretion via type II machinery by a second messenger signaling system.

Though we do not know the exact post-translational mechanism of regulation, existing evidence from our laboratory and others enables us to speculate a variety of possibilities. Given that the T2SS secreton is polar-localized [26] in *P*. *aeruginosa*, it is likely that the effect of c-di-GMP turnover by MorA is local and the signal is transmitted directly to the secretion machinery i.e. at the level of secretion efficiency. This notion of spatial sequestration of c-di-GMP pools for regulation of specific cellular functions is well-known in many bacterial species [51, 80–85]. It is possible that changes in c-di-GMP levels may directly i) alter the activity of inner membrane transport machineries, *sec* and/or *tat* systems; ii) increase the efficiency of ATPase-mediated pseudopilin activity (pushing out the periplasmic proteins through outer membrane ring) or iii) control the periplasmic processing of secreted proteins as in the case of LapD [52, 86]. On the contrary, MorA signaling might have an indirect effect on T2SS through a signaling cascade in association with similar proteins [84, 85] or crosstalk with other regulatory virulence systems that control secretion, such as the pyoverdine system that regulates PrpL [72] and the quorum sensing system as in the case of TbpB in *P*. *aeruginosa* [87].

Further investigations are required to understand the mechanism by which MorA-cyclic-di-GMP signaling affects secretion. However, c-di-GMP receptors known are very eclectic in nature [47, 51, 88–91], it is difficult to predict possible intermediary players unless experimentally validated. Nevertheless, proteins with predicted c-di-GMP binding canonical motifs can be used to test their roles as receptors in this signaling process. Alternatively, cytoplasmic membrane-localized T2SS structural proteins could also be targeted to test c-di-GMP binding efficiency and/or any post-translational modification.

## Supporting Information

S1 Fig Standard curve for elastase activity. (PDF)

S2 Fig MALDI-ToF-ToF spectra of secreted proteins affected by MorA. (PDF)

S3 Fig RNA levels of major secreted proteases show no significant change due to MorA. (PDF)

S1 Table List of primers used in this study. (PDF)

S2 Table MALDI-ToF-ToF identification of *P*. *aeruginosa* secreted proteins affected by MorA. (PDF)

## References

[1] Lyczak JB, Cannon CL, Pier GB. Establishment of *Pseudomonas aeruginosa* infection: lessons from a versatile opportunist. Microbes Infect. 2000;2: 1051–1060. 10967285 10.1016/s1286-4579(00)01259-4

[2] Fleiszig SMJ, Wiener-Kronish JP, Miyazaki H, Vallas V, Mostov KE, Kanda D, et al *Pseudomonas aeruginosa*-mediated cytotoxicity and invasion correlate to distinct genotypes at the loci encoding exoenzyme S. Infect Immun. 1997;65: 579–586. 9009316 10.1128/iai.65.2.579-586.1997 PMC176099

[3] Fleiszig SMJ, Wiener-Kronish JP, Vallas V, Mostov KE, Frank DW. Evidence that all *Pseudomonas aeruginosa* strains may be inherently capable of invading corneal epithelial cells and that cytotoxicity is regulated by ExsA. ARVO Abstr Investig Ophthalmol Vis Sci. 1996;37: S0.

[4] Pollack M. *Pseudomonas aeruginosa* In: Mandell GL, Bennett JE, Dolin R, editors. Principles and Practice of Infectious Diseases. 5th ed New York, NY: Churchill Livingstone; 2000 p. 2310–2327.

[5] Fleiszig SMJ, Zaidi TS, Pier GB. *Pseudomonas aeruginosa* survival and multiplication within corneal epithelial cells *in vitro* . Infect Immun. 1995;63: 4072–4077. 7558321 10.1128/iai.63.10.4072-4077.1995 PMC173572

[6] Massengale AR, Quinn FJ, Williams A, Gallagher S, Aronoff SC. The effect of alginate on the invasion of cystic fibrosis respiratory epithelial cells by clinical isolates of *Pseudomonas aeruginosa* . Exp Lung Res. 2000;26: 163–178. 10813089 10.1080/019021400269853

[7] Saier MH Jr. Protein secretion and membrane insertion systems in gram-negative bacteria. J Membr Biol. 2006;4: 75–90. 10.1007/s00232-006-0049-7 17546510

[8] Bleves S, Viarre V, Salacha R, Michel GP, Filloux A, Voulhoux R. Protein secretion systems in *Pseudomonas aeruginosa*: A wealth of pathogenic weapons. Int J Med Microbiol. 2010;300: 534–543. 10.1016/j.ijmm.2010.08.005 20947426

[9] He SY, Schoedel C, Chatterjee AK, Collmer A. Extracellular secretion of pectate lyase by the *Erwinia chrysanthemi* out pathway is dependent upon Sec-mediated export across the inner membrane. J Bacteriol. 1991;173: 4310–4317. 1829728 10.1128/jb.173.14.4310-4317.1991 PMC208090

[10] Voulhoux R, Ball G, Ize B, Vasil ML, Lazdunski A, Wu LF, et al Involvement of the twin-arginine translocation system in protein secretion via the type II pathway. EMBO J. 2001;20: 6735–6741. 11726509 10.1093/emboj/20.23.6735 PMC125745

[11] Sandkvist M. Biology of type II secretion. Mol Microbiol. 2001;40: 271–283. 11309111 10.1046/j.1365-2958.2001.02403.x

[12] Sandkvist M. Type II secretion and pathogenesis. Infect Immun. 2001;69: 3523–3535. 11349009 10.1128/IAI.69.6.3523-3535.2001 PMC98326

[13] von Tils D, Blädel I, Schmidt MA, Heusipp G. Type II secretion in *Yersinia*- a secretion system for pathogenicity and environmental fitness. Front Cell Infect Microbiol. 2012;2: 160 10.3389/fcimb.2012.00160 23248779 PMC3521999

[14] Cianciotto NP. Type II secretion and *Legionella* virulence. Curr Top Microbiol Immunol. 2013;376: 81–102. 10.1007/82_2013_339 23900831

[15] Johnson TL, Fong JC, Rule C, Rogers A, Yildiz FH, Sandkvist M. The Type II secretion system delivers matrix proteins for biofilm formation by *Vibrio cholerae* . J Bacteriol. 2014;196: 4245–4252. 10.1128/JB.01944-14 25266381 PMC4248848

[16] Michel GPF, Voulhoux R. The Type II Secretory System (T2SS) in Gram-negative Bacteria: A Molecular Nanomachine for Secretion of Sec and Tat-Dependent Extracellular Proteins, In: Wooldridge K, editor. Bacterial Secreted Proteins: Secretory Mechanisms and Role in Pathogenesis. Norfolk, UK: Caister Academic Press; 2009 p. 67–92.

[17] Reichow SL, Korotkov KV, Hol WG, Gonen T. Structure of the cholera toxin secretion channel in its closed state. Nat Struct Mol Biol. 2010;17: 1226–1232. 10.1038/nsmb.1910 20852644 PMC2950906

[18] Korotkov KV, Sandkvist M, Hol WG. The type II secretion system: biogenesis, molecular architecture and mechanism. Nat Rev Microbiol. 2012;10: 336–351. 10.1038/nrmicro2762 22466878 PMC3705712

[19] Douzi B, Filloux A, Voulhoux R. On the path to uncover the bacterial type II secretion system. Philos Trans R Soc Lond B Biol Sci. 2012;367: 1059–1072. 10.1098/rstb.2011.0204 22411978 PMC3297435

[20] Nivaskumar M, Francetic O. Type II secretion system: a magic beanstalk or a protein escalator. Biochim Biophys Acta. 2014;1843: 1568–1577. 10.1016/j.bbamcr.2013.12.020 24389250

[21] Martínez A, Ostrovsky P, Nunn DN. Identification of an additional member of the secretin superfamily of proteins in *Pseudomonas aeruginosa* that is able to function in type II protein secretion. Mol Microbiol. 1998;28: 1235–1246. 9680212 10.1046/j.1365-2958.1998.00888.x

[22] Ball G, Durand E., Lazdunski A., Filloux A. A novel type II secretion system in *Pseudomonas aeruginosa* . Mol Microbiol. 2002;43: 475–485. 11985723 10.1046/j.1365-2958.2002.02759.x

[23] Michel GPF, Durand E, Filloux A. XphA/XqhA, a novel GspCD subunit for type II secretion in *Pseudomonas aeruginosa* . J Bacteriol. 2007;189: 3776–3783. 17351035 10.1128/JB.00205-07 PMC1913328

[24] Filloux A, Michel G, Bally M. GSP-dependent protein secretion in gram-negative bacteria: the Xcp system of *Pseudomonas aeruginosa* . FEMS Microbiol Rev. 1998;22: 177–198. 9818381 10.1111/j.1574-6976.1998.tb00366.x

[25] Brok R, Van Gelder P, Winterhalter M, Ziese U, Koster AJ, de Cock H, et al The C-terminal domain of the *Pseudomonas* secretin XcpQ forms oligomeric rings with pore activity. J Mol Biol. 1999;294: 1169–1179. 10600375 10.1006/jmbi.1999.3340

[26] Senf F, Tommassen J, Koster M. Polar secretion of proteins via the Xcp type II secretion system in *Pseudomonas aeruginosa* . Microbiol. 2008;154: 3025–3032. 10.1099/mic.0.2008/018069-0 18832308

[27] Filloux A. The underlying mechanisms of type II protein secretion. Biochim Biophys Acta. 2004;1694: 163–179. 15546665 10.1016/j.bbamcr.2004.05.003

[28] McIver KS, Kessler E, Olson JC, Ohman DE. The elastase propeptide functions as an intramolecular chaperone required for elastase activity and secretion in *Pseudomonas aeruginosa* . Mol Microbiol. 1995;18: 877–889. 8825092 10.1111/j.1365-2958.1995.18050877.x

[29] Lu HM, Lory S. A specific targeting domain in mature exotoxin A is required for its extracellular secretion from *Pseudomonas aeruginosa* . EMBO J. 1996;15: 429–436. 8617218 PMC449958

[30] Palomäki T, Pickersgill R, Riekki R, Romantschuk M, Saarilahti HT. A putative three-dimensional targeting motif of polygalacturonase (PehA), a protein secreted through the type II (GSP) pathway in *Erwinia carotovora* . Mol Microbiol. 2002;43: 585–596. 11929517 10.1046/j.1365-2958.2002.02793.x

[31] Stover CK, Pham XQ, Erwin AL, Mizoguchi SD, Warrener P, Hickey MJ, et al Complete genome sequence of *Pseudomonas aeruginosa* PAO1, an opportunistic pathogen. Nature. 2000;406: 959–964. 10984043 10.1038/35023079

[32] Smith RS, Iglewski BH. *P*. *aeruginosa* quorum-sensing systems and virulence. Curr Opin Microbiol. 2003;6: 56–60. 12615220 10.1016/s1369-5274(03)00008-0

[33] Whiteley M, Lee KM, Greenberg EP. Identification of genes controlled by quorum sensing in *Pseudomonas aeruginosa* . Proc Natl Acad Sci U S A. 1999;96: 13904–13909. 10570171 10.1073/pnas.96.24.13904 PMC24163

[34] Erickson DL, Endersby R, Kirkham A, Stuber K, Vollman DD, Rabin HR, et al *Pseudomonas aeruginosa* quorum-sensing systems may control virulence factor expression in the lungs of patients with cystic fibrosis. Infect Immun. 2002;70: 1783–1790. 11895939 10.1128/IAI.70.4.1783-1790.2002 PMC127834

[35] Chapon-Hervé V, Akrim M, Latifi A, Williams P, Lazdunski A, Bally M. Regulation of the xcp secretion pathway by multiple quorum-sensing modulons in *Pseudomonas aeruginosa* . Mol Microbiol. 1997;24: 1169–1178. 9218766 10.1046/j.1365-2958.1997.4271794.x

[36] Schuster M, Lostroh CP, Ogi T, Greenberg EP. Identification, timing, and signal specificity of *Pseudomonas aeruginosa* quorum-controlled genes: a transcriptome analysis. J Bacteriol. 2003;185: 2066–2079. 12644476 10.1128/JB.185.7.2066-2079.2003 PMC151497

[37] Wagner VE, Bushnell D, Passador L, Brooks AI, Iglewski BH. Microarray analysis of *Pseudomonas aeruginosa* quorum-sensing regulons: effects of growth phase and environment. J Bacteriol. 2003;185: 2080–2095. 12644477 10.1128/JB.185.7.2080-2095.2003 PMC151498

[38] Nouwens AS, Beatson SA, Whitchurch CB, Walsh BJ, Schweizer HP, Mattick JS, et al Proteome analysis of extracellular proteins regulated by the las and rhl quorum sensing systems in *Pseudomonas aeruginosa* PAO1. Microbiol. 2003;149: 1311–1322. 12724392 10.1099/mic.0.25967-0

[39] Albus AM, Pesci EC, Runyen-Janecky LJ, West SE, Iglewski BH. Vfr controls quorum sensing in *Pseudomonas aeruginosa* . J Bacteriol. 1997;179: 3928–3935. 9190808 10.1128/jb.179.12.3928-3935.1997 PMC179201

[40] Cotter PA, Stibitz S. c-di-GMP-mediated regulation of virulence and biofilm formation. Curr Opin Microbiol. 2007;10: 17–23. 17208514 10.1016/j.mib.2006.12.006

[41] Tamayo R, Pratt JT, Camilli A. Roles of cyclic diguanylate in the regulation of bacterial pathogenesis. Annu Rev Microbiol. 2007;61: 131–148. 17480182 10.1146/annurev.micro.61.080706.093426 PMC2776827

[42] Römling U, Simm R. Prevailing concepts of c-di-GMP signaling In: Collin M, Schuch R, editors. Bacterial sensing and signaling. Basel, Switzerland: Karger Contrib Microbiol. 2009;16: 161–181. 10.1159/000219379 19494585

[43] Simm R, Morr M, Kader A, Nimtz M, Römling U. GGDEF and EAL domains inversely regulate cyclic di-GMP levels and transition from sessility to motility. Mol Microbiol. 2004;53: 1123–1134. 15306016 10.1111/j.1365-2958.2004.04206.x

[44] D'Argenio DA, Calfee MW, Rainey PB, Pesci EC. Autolysis and autoaggregation in *Pseudomonas aeruginosa* colony morphology mutants. J Bacteriol. 2002;184: 6481–6489. 12426335 10.1128/JB.184.23.6481-6489.2002 PMC135425

[45] Borlee BR, Goldman AD, Murakami K, Samudrala R, Wozniak DJ, Parsek MR. *Pseudomonas aeruginosa* uses a cyclic-di-GMP-regulated adhesin to reinforce the biofilm extracellular matrix. Mol Microbiol. 2010;75: 827–842. 10.1111/j.1365-2958.2009.06991.x 20088866 PMC2847200

[46] Kulasakara H, Lee V, Brencic A, Liberati N, Urbach J, Miyata S, et al Analysis of *Pseudomonas aeruginosa* diguanylate cyclases and phosphodiesterases reveals a role for bis-(3'-5')- cyclic-GMP in virulence. Proc Natl Acad Sci U S A. 2006;103: 2839–2844. 16477007 10.1073/pnas.0511090103 PMC1413825

[47] Weber H, Pesavento C, Possling A, Tischendorf G, Hengge R. Cyclic-di- GMP-mediated signalling within the sigma network of *Escherichia coli* . Mol Microbiol. 2006;62: 1014–1034. 17010156 10.1111/j.1365-2958.2006.05440.x

[48] Levi A, Jenal U. Holdfast formation in motile swarmer cells optimizes surface attachment during *Caulobacter crescentus* development. J Bacteriol. 2006;188: 5315–5318. 16816207 10.1128/JB.01725-05 PMC1539976

[49] Kader A, Simm R, Gerstel U, Morr M, Römling U. Hierarchical involvement of various GGDEF domain proteins in rdar morphotype development of *Salmonella enterica serovar Typhimurium* . Mol Microbiol. 2006;60: 602–616. 16629664 10.1111/j.1365-2958.2006.05123.x

[50] Sudarsan N, Lee E R, Weinberg Z, Moy RH, Kim JN, Link KH, et al Riboswitches in eubacteria sense the second messenger cyclic di-GMP. Science. 2008;3: 411–413. 10.1126/science.1159519 PMC5304454 18635805

[51] Tuckerman JR, Gonzalez G, Gilles-Gonzalez MA. Cyclic di-GMP activation of polynucleotide phosphorylase signal-dependent RNA processing. J Mol Biol. 2011;407: 633–639. 10.1016/j.jmb.2011.02.019 21320509

[52] Navarro MV, Newell PD, Krasteva PV, Chatterjee D, Madden DR, O’Toole GA, et al Structural basis for c-di-GMP-mediated inside-out signaling controlling periplasmic proteolysis. PLoS Biol. 2011;9: e1000588 10.1371/journal.pbio.1000588 21304926 PMC3032553

[53] Moscoso JA, Mikkelsen H, Heeb S, Williams P, Filloux A. The *Pseudomonas aeruginosa* sensor RetS switches type III and type VI secretion via c-di-GMP signalling. Environ Microbiol. 2011;13: 3128–3138. 10.1111/j.1462-2920.2011.02595.x 21955777

[54] Pérez-Mendoza D, Coulthurst SJ, Humphris S, Campbell E, Welch M, Toth IK, et al A multi-repeat adhesin of the phytopathogen, *Pectobacterium atrosepticum*, is secreted by a Type I pathway and is subject to complex regulation involving a non-canonical diguanylate cyclase. Mol Microbiol. 2011;82: 719–733. 10.1111/j.1365-2958.2011.07849.x 21992096

[55] Sheng L, Lv Y, Liu Q, Wang Q, Zhang Y. Connecting type VI secretion, quorum sensing, and c-di-GMP production in fish pathogen *Vibrio alginolyticus* through phosphatase PppA. Vet Microbiol. 2013;162: 652–662. 10.1016/j.vetmic.2012.09.009 23021863

[56] Choy WK, Zhou L, Syn CK, Zhang LH, Swarup S. MorA defines a new class of regulators affecting flagellar development and biofilm formation in diverse *Pseudomonas* species. J Bacteriol. 2004;186: 7221–7228. 15489433 10.1128/JB.186.21.7221-7228.2004 PMC523210

[57] Choy W, Bajic V, Heng M, Veronika M, Swarup S. Regulatory networks of genes affected by MorA, a global regulator containing GGDEF and EAL domains in *Pseudomonas aeruginosa* In: Leong HW, Sung W-K, Esking E, editors. Series on Advances in Bioinformatics and Computational Biology. Regulatory Genomics. Proceedings of the 3^rd^ annual RECOMB workshop: Vol 8; 2006 7 17–18; Singapore: World Scientific; 2008 p. 123–129.

[58] Meissner A, Wild V, Simm R, Rohde M, Erck C, Bredenbruch F, et al *Pseudomonas aeruginosa* cupA-encoded fimbriae expression is regulated by a GGDEF and EAL domain-dependent modulation of the intracellular level of cyclic diguanylate. Environ Microbiol. 2007;9: 2475–2485. 17803773 10.1111/j.1462-2920.2007.01366.x

[59] Phippen CW, Mikolajek H, Schlaefli HG, Keevil CW, Webb JS, Tews I. Formation and dimerization of the phosphodiesterase active site of the *Pseudomonas aeruginosa* MorA, a bi-functional c-di-GMP regulator. FEBS Lett. 2014;588: 4631–4636. 10.1016/j.febslet.2014.11.002 25447517

[60] Dasgupta N, Wolfgang MC, Goodman AL, Arora SK, Jyot J, Lory S, et al A four-tiered transcriptional regulatory circuit controls flagellar biogenesis in *Pseudomonas aeruginosa* . Mol Microbiol. 2003;50: 809–824. 14617143 10.1046/j.1365-2958.2003.03740.x

[61] Schäfer A, Tauch A, Jäger W, Kalinowski J, Thierbach G, Pühler A. Small mobilizable multi-purpose cloning vectors derived from the Escherichia coli plasmids pK18 and pK19: Selection of defined deletions in the chromosome of *Corynebacterium glutamicum* . Gene. 1994;145: 69–73. 8045426 10.1016/0378-1119(94)90324-7

[62] Figurski DH, Helinski DR. Replication of an origin-containing derivative of plasmid RK2 dependent on a plasmid function provided *in trans* . Proc Natl Acad Sci USA. 1979;76: 1648–1652. 377280 10.1073/pnas.76.4.1648 PMC383447

[63] Ha UH, Kim J, Badrane H, Jia J, Baker HV, Wu D, et al An in vivo inducible gene of *Pseudomonas aeruginosa* encodes an anti-ExsA to suppress the type III secretion system. Mol Microbiol. 2004;54: 307–320. 15469505 10.1111/j.1365-2958.2004.04282.x

[64] Morihara K, Tsuzuki H, Oka T, Inoue H, Ebata M. *Pseudomonas aeruginosa* elastase. Isolation, crystallization, and preliminary characterization. J Biol Chem. 1965;240: 3295–3304. 14321366

[65] Robert V, Filloux A, Michel GPF. Subcomplexes from the Xcp secretion system of *Pseudomonas aeruginosa* . FEMS Microbiol Lett. 2005;252: 43–50. 16168578 10.1016/j.femsle.2005.08.029

[66] Robert V, Filloux A, Michel GPF. Role of XcpP in the functionality of the *Pseudomonas aeruginosa* secreton. Res Microbiol. 2005;156: 880–886. 15936176 10.1016/j.resmic.2005.04.002

[67] Fleiszig SM, Arora SK, Van R, Ramphal R. FlhA, a component of the flagellum assembly apparatus of *Pseudomonas aeruginosa*, plays a role in internalization by corneal epithelial cells. Infect Immun. 2001;69: 4931–4937. 11447170 10.1128/IAI.69.8.4931-4937.2001 PMC98584

[68] Folders J, Tommassen J, van Loon LC, Bitter W. Identification of a chitin- binding protein secreted by *Pseudomonas aeruginosa* . J Bacteriol. 2009;182: 1257–1263. 10.1016/j.juro.2009.07.071 10671445 PMC94410

[69] Braun P, de Groot A, Bitter W, Tommassen J. Secretion of elastinolytic enzymes and their propeptides by *Pseudomonas aeruginosa* . J Bacteriol. 1998;180: 3467–3469. 9642203 10.1128/jb.180.13.3467-3469.1998 PMC107305

[70] Wilderman PJ, Vasil AI, Johnson Z, Wilson MJ, Cunliffe HE, Lamont IL, et al Characterization of an endoprotease (PrpL) encoded by a PvdS-regulated gene in *Pseudomonas aeruginosa* . Infect Immun. 2001;69: 5385–5394. 11500408 10.1128/IAI.69.9.5385-5394.2001 PMC98648

[71] Cowell BA, Twining SS, Hobden JA, Kwong MSF, Fleiszig SMJ. Mutation of lasA and lasB reduces *Pseudomonas aeruginosa* invasion of epithelial cells. Microbiol. 2003;149: 2291–2299. 10.1099/mic.0.26280-0 12904569

[72] Engel LS, Hill JM, Caballero AR, Green LC, O'Callaghan RJ. Protease IV, a unique extracellular protease and virulence factor from *Pseudomonas aeruginosa* . J Biol Chem. 1998;273: 16792–16797. 9642237 10.1074/jbc.273.27.16792

[73] Jacobs MA, Alwood A, Thaipisuttikul I, Spencer D, Haugen E, Ernst S, et al Comprehensive transposon mutant library of *Pseudomonas aeruginosa* . Proc Natl Acad Sci U S A. 2003;100: 14339–14344. 14617778 10.1073/pnas.2036282100 PMC283593

[74] Bitter W, Koster M, Latijnhouwers M, de Cock H, Tommassen J. Formation of oligomeric rings by XcpQ and PilQ, which are involved in protein transport across the outer membrane of *Pseudomonas aeruginosa* . Mol Microbiol. 1998;27: 209–219. 9466268 10.1046/j.1365-2958.1998.00677.x

[75] Grande KK, Gustin JK, Kessler E, Ohman DE. Identification of critical residues in the propeptide of LasA protease of *Pseudomonas aeruginosa* involved in the formation of a stable mature protease. J Bacteriol. 2007;189: 3960–3968. 17351039 10.1128/JB.01828-06 PMC1913401

[76] Cahan R, Axelrad I, Safrin M, Ohman DE, Kessler E. A secreted aminopeptidase of *Pseudomonas aeruginosa*. Identification, primary structure, and relationship to other aminopeptidases. J Biol Chem. 2001;276: 43645–43652. 11533066 10.1074/jbc.M106950200

[77] Matsumoto K. Role of bacterial proteases in pseudomonal and serratial keratitis. Biol Chem. 2004;385: 1007–1016. 15576320 10.1515/BC.2004.131

[78] Traidej M, Caballero AR, Marquart ME, Thibodeaux BA, O'Callaghan RJ. Molecular analysis of *Pseudomonas aeruginosa* protease IV expressed in *Pseudomonas putida* . Invest Ophthalmol Vis Sci. 2003;44: 190–196. 12506074 10.1167/iovs.02-0458

[79] Bauman SJ, Kuehn MJ. Purification of outer membrane vesicles from *Pseudomonas aeruginosa* and their activation of an IL-8 response. Microbes Infect. 2006;8: 2400–2408. 16807039 10.1016/j.micinf.2006.05.001 PMC3525494

[80] Paul R, Weiser S, Amiot NC, Chan C, Schirmer T, et al Cell cycle-dependent dynamic localization of a bacterial response regulator with a novel di-guanylate cyclase output domain. Genes Dev. 2004;18: 715–727. 15075296 10.1101/gad.289504 PMC387245

[81] Guvener ZT, Harwood CS. Subcellular location characteristics of the *Pseudomonas aeruginosa* GGDEF protein, WspR, indicate that it produces cyclic-di-GMP in response to growth on surfaces. Mol Microbiol. 2007;66: 1459–1473. 18028314 10.1111/j.1365-2958.2007.06008.x PMC4105145

[82] Merritt JH, Ha DG, Cowles KN, Lu W, Morales DK, Rabinowitz J, et al Specific control of *Pseudomonas aeruginosa* surface-associated behaviors by two c-di-GMP diguanylate cyclases. MBio. 2010;1: e00183–10. doi: 10.1128/ mBio.00183-10 20978535 10.1128/mBio.00183-10 PMC2957078

[83] Christen M, Kulasekara HD, Christen B, Kulasekara BR, Hoffman LR, Miller SI. Asymmetrical distribution of the second messenger c-di-GMP upon bacterial cell division. Science. 2010;328: 1295–1297. 10.1126/science.1188658 20522779 PMC3906730

[84] Abel S, Chien P, Wassmann P, Schirmer T, Kaever V, Laub MT, et al Regulatory cohesion of cell cycle and cell differentiation through interlinked phosphorylation and second messenger networks. Mol Cell. 2011;43: 550–560. 10.1016/j.molcel.2011.07.018 21855795 PMC3298681

[85] Lindenberg S, Klauck G, Pesavento C, Klauck E, Hengge R. The EAL domain protein YciR acts as a trigger enzyme in a c-di-GMP signalling cascade in *E*. *coli* biofilm control. EMBO J. 2013;32: 2001–2014. 10.1038/emboj.2013.120 23708798 PMC3715855

[86] Newell PD, Boyd CD, Sondermann H, O'Toole GA. A c-di-GMP effector system controls cell adhesion by inside-out signaling and surface protein cleavage. PLoS Biol. 2011;9: e1000587 10.1371/journal.pbio.1000587 21304920 PMC3032545

[87] Ueda A, Wood TK. Connecting Quorum Sensing, c-di-GMP, Pel Polysaccharide, and Biofilm Formation in *Pseudomonas aeruginosa* through Tyrosine Phosphatase TpbA (PA3885). PLoS Pathog. 2009;5: e1000483 10.1371/journal.ppat.1000483 19543378 PMC2691606

[88] Ryjenkov DA, Simm R, Römling U, Gomelsky M. The PilZ domain is a receptor for the second messenger c-di-GMP: the PilZ domain protein YcgR controls motility in enterobacteria. J Biol Chem. 2006;281: 30310–30314. 16920715 10.1074/jbc.C600179200

[89] Lee VT, Matewish JM, Kessler JL, Hyodo M, Hayakawa Y, Lory S. A cyclic-di-GMP receptor required for bacterial exopolysaccharide production. Mol Microbiol. 2007;65: 1474–1484. 17824927 10.1111/j.1365-2958.2007.05879.x PMC2170427

[90] Tao F, He YW, Wu D.H, Swarup S, Zhang LH. The cyclic nucleotide monophosphate domain of *Xanthomonas campestris* global regulator Clp defines a new class of cyclic di-GMP effectors. J Bacteriol. 2010;192: 1020–1029. 10.1128/JB.01253-09 20008070 PMC2812978

[91] Pratt JT, Tamayo R, Tischler AD, Camilli A. PilZ domain proteins bind cyclic diguanylate and regulate diverse processes in *Vibrio cholerae* . J Biol Chem. 2007;282: 12860–12870. 17307739 10.1074/jbc.M611593200 PMC2790426

