# Supplementary material for: Global Regulator MorA Affects Virulence-Associated Protease Secretion in Pseudomonas aeruginosa PAO1
Source: PLoS One. 2015 Apr 20;10(4):e0123805. doi: 10.1371/journal.pone.0123805 (PMC4404142; doi:10.1371/journal.pone.0123805)
Supplement: S1 Fig — (PDF) [file pone.0123805.s001.pdf]

Global Regulator MorA affects Virulence-associated Protease Secretion in *Pseudomonas aeruginosa* PAO1

Supporting Information

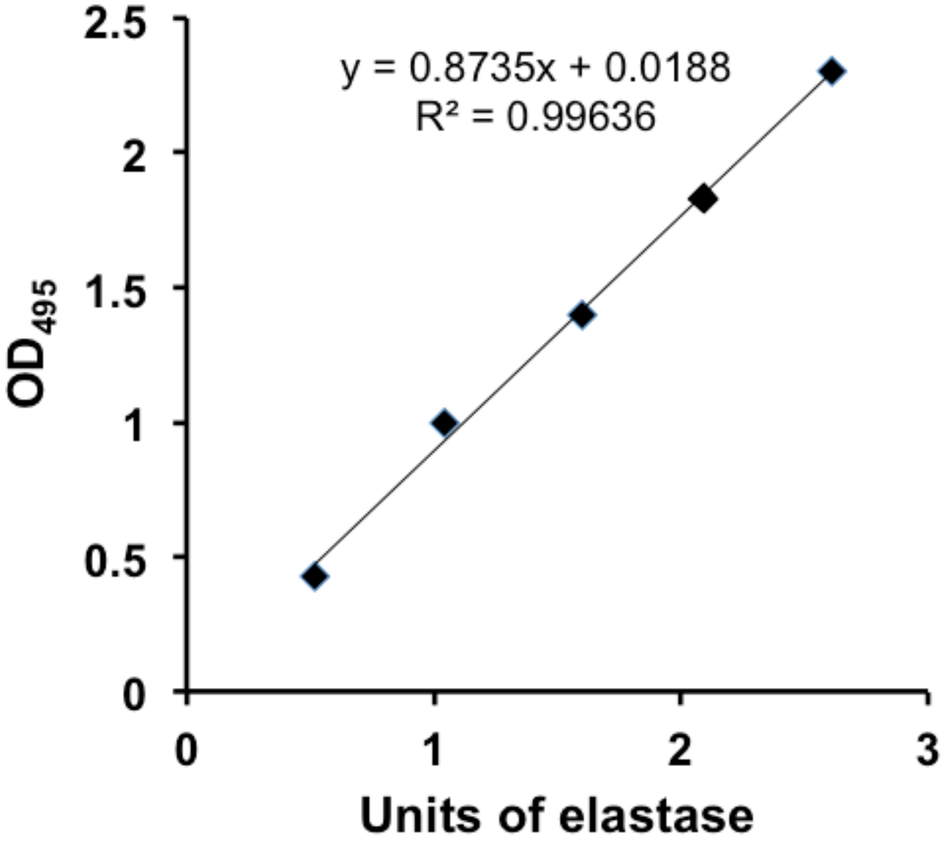

S1 Figure. Standard curve for elastase activity. Activity measured with increasing units of *Pseudomonas aeruginosa* elastase on elastin congo-red (Sigma). Reaction buffer without elastase was used as blank. At end of 6 hr incubation, the absorbance of the supernatant containing cleaved congo-red was measured at 495nm.
