# Supplementary material for: Global Regulator MorA Affects Virulence-Associated Protease Secretion in Pseudomonas aeruginosa PAO1
Source: PLoS One. 2015 Apr 20;10(4):e0123805. doi: 10.1371/journal.pone.0123805 (PMC4404142; doi:10.1371/journal.pone.0123805)

Global Regulator MorA affects Virulence-associated Protease Secretion in *Pseudomonas aeruginosa* PAO1

Supporting Information

S2 Figure. MALDI-ToF-ToF spectra of secreted proteins affected by MorA

A. **PA0752**

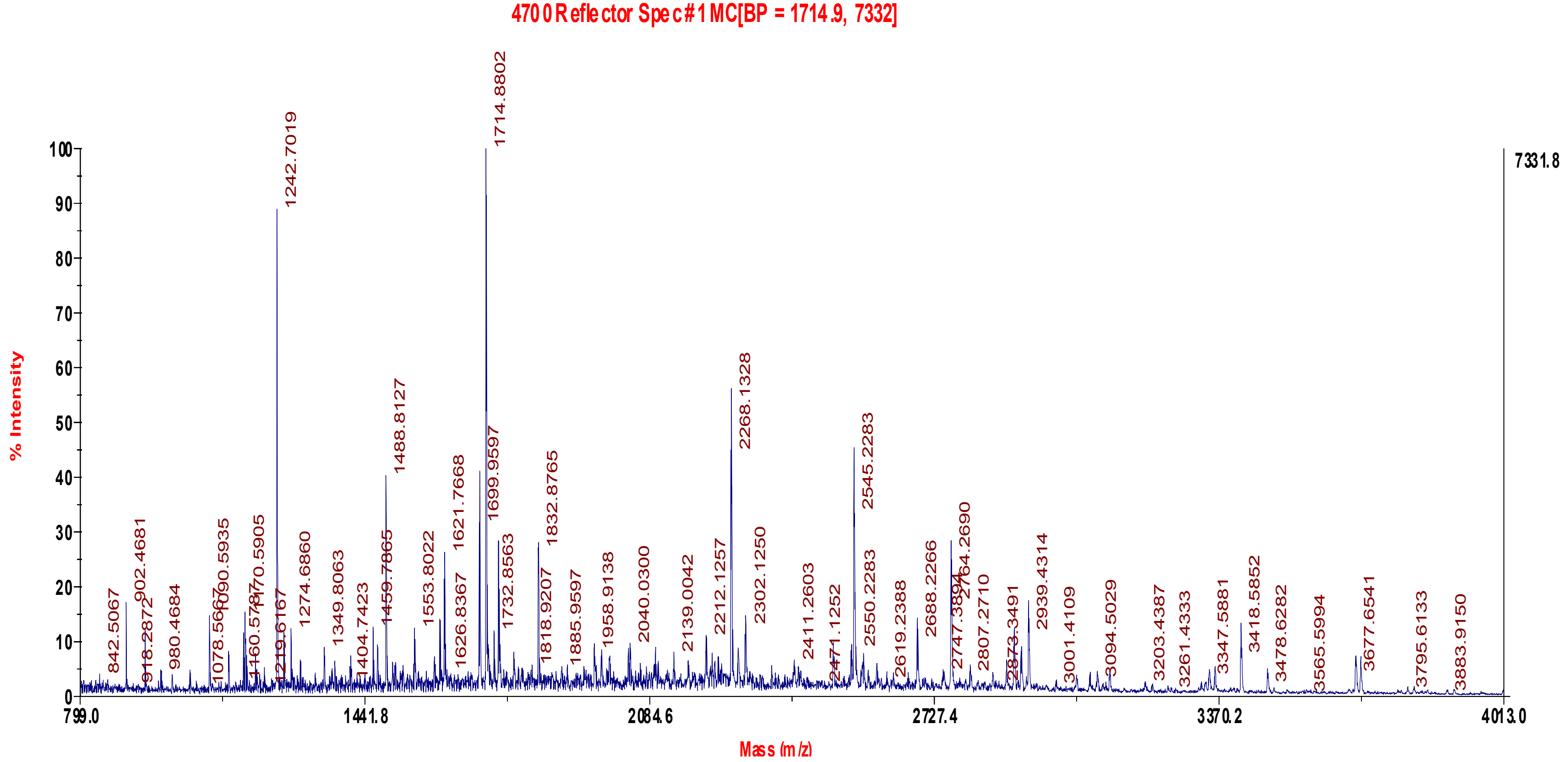

B. Probable aminopeptidase

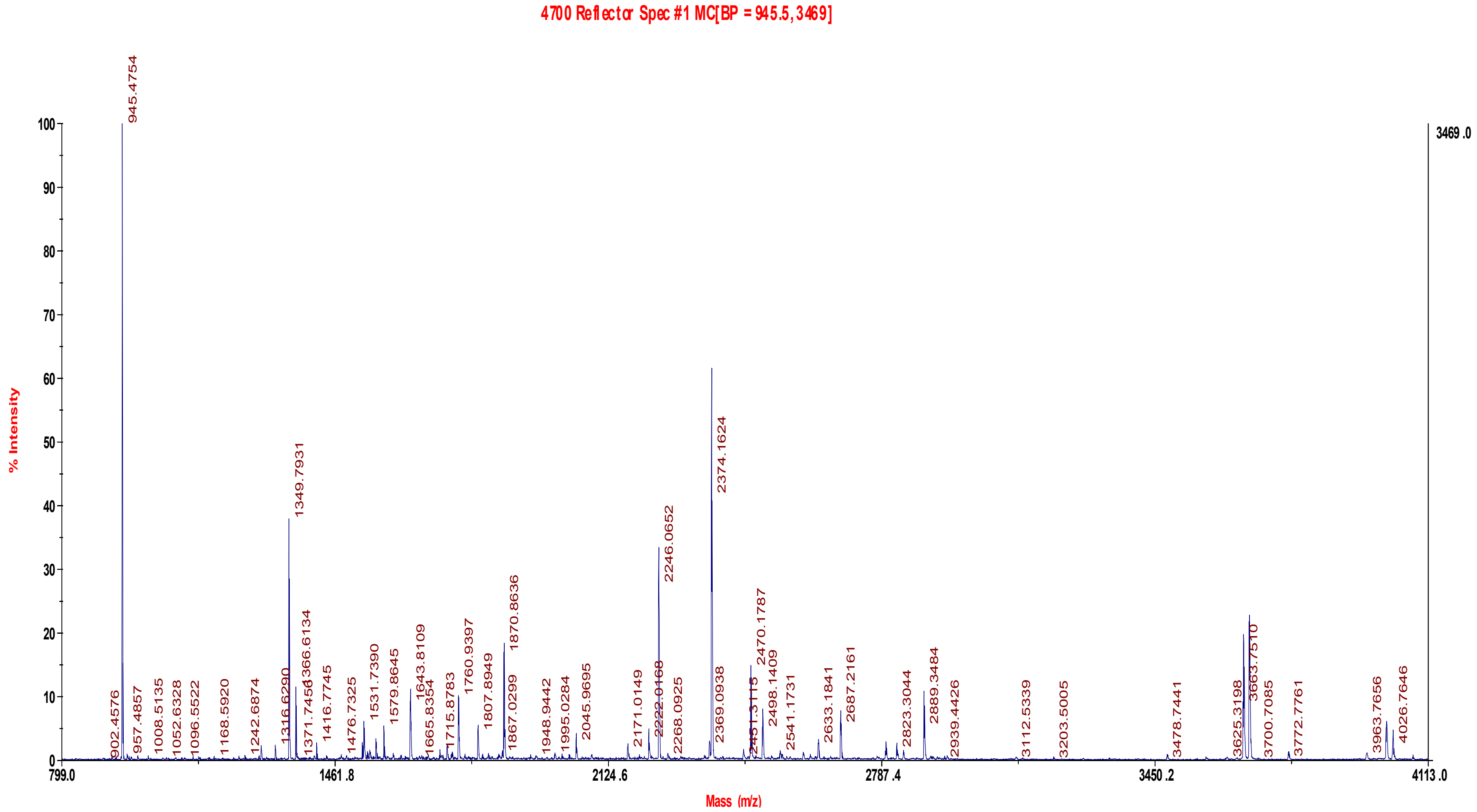

C. **Alkaline metalloproteinase precursor AprA**

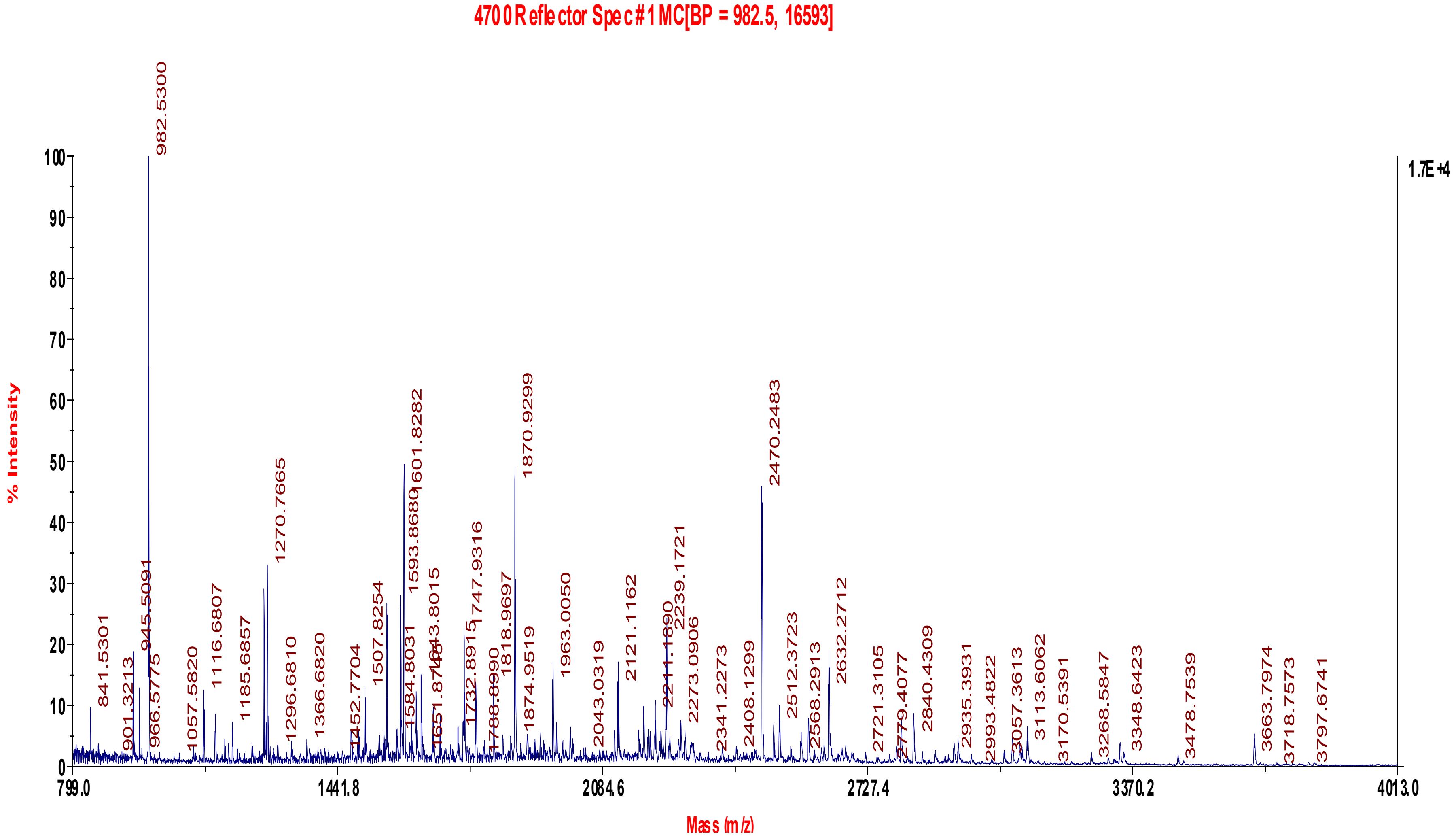

D. Chitin binding protein CbpD

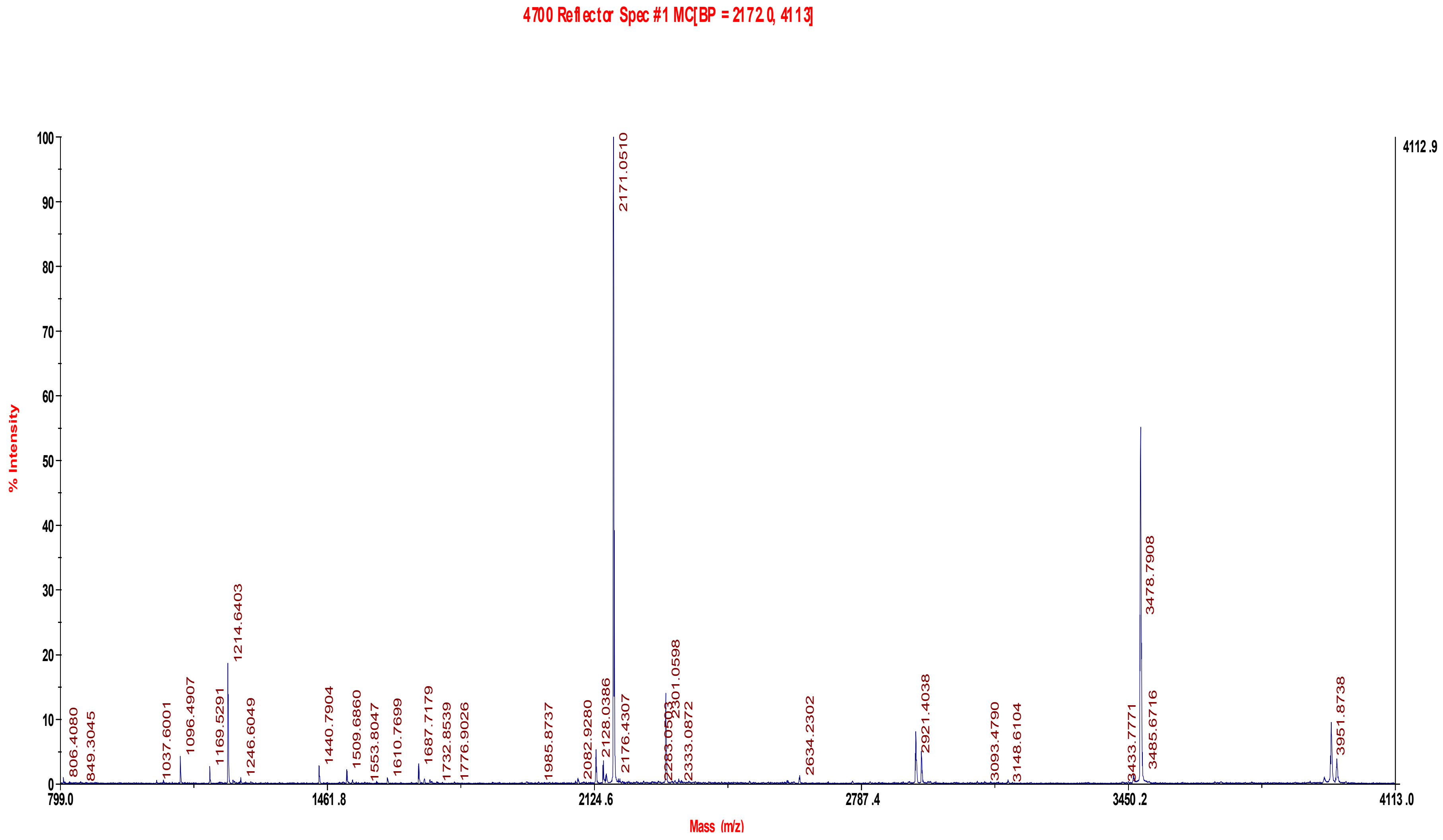

E. Elastase LasB

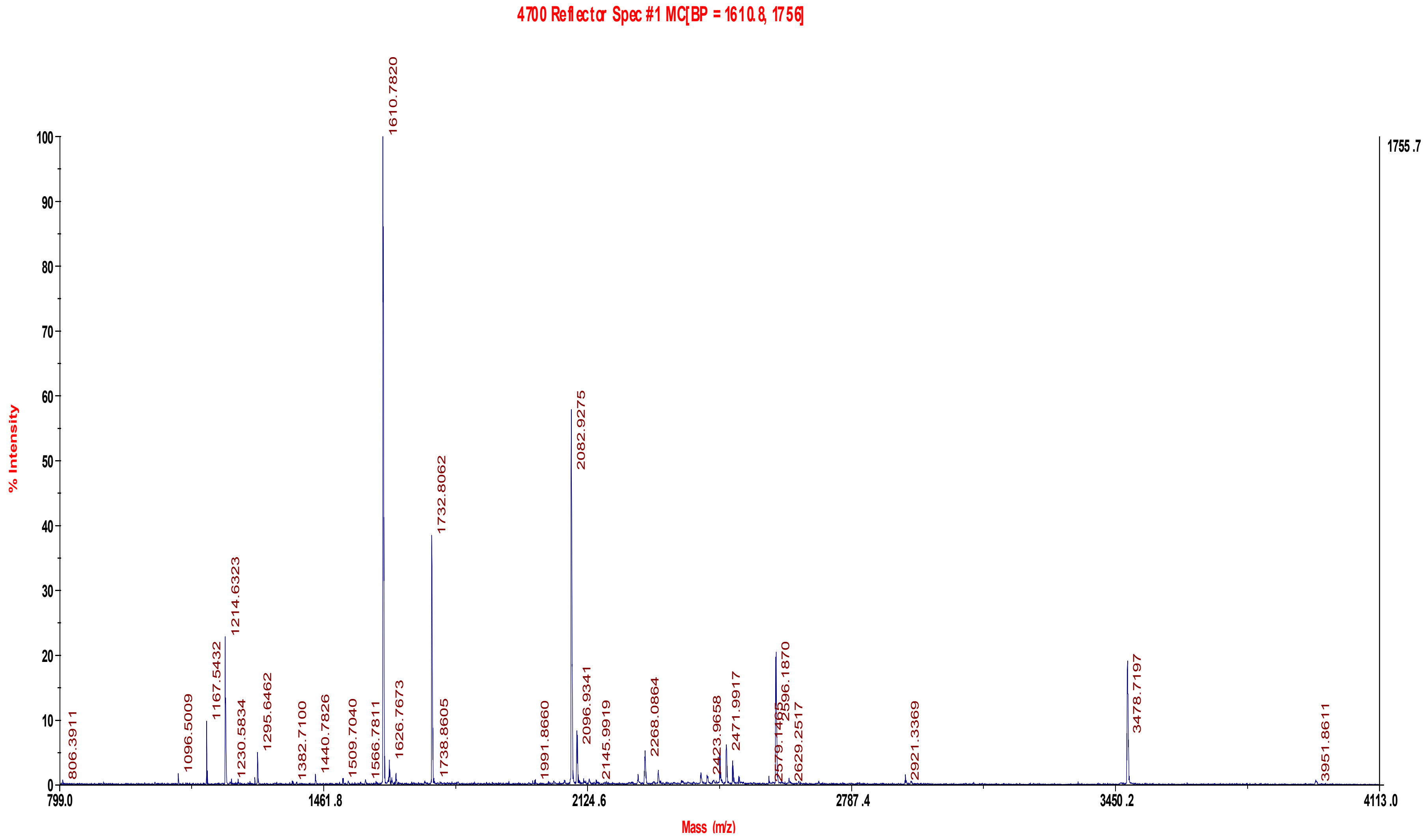

F. Pvds-regulated endoprotease; lysyl class PrpL

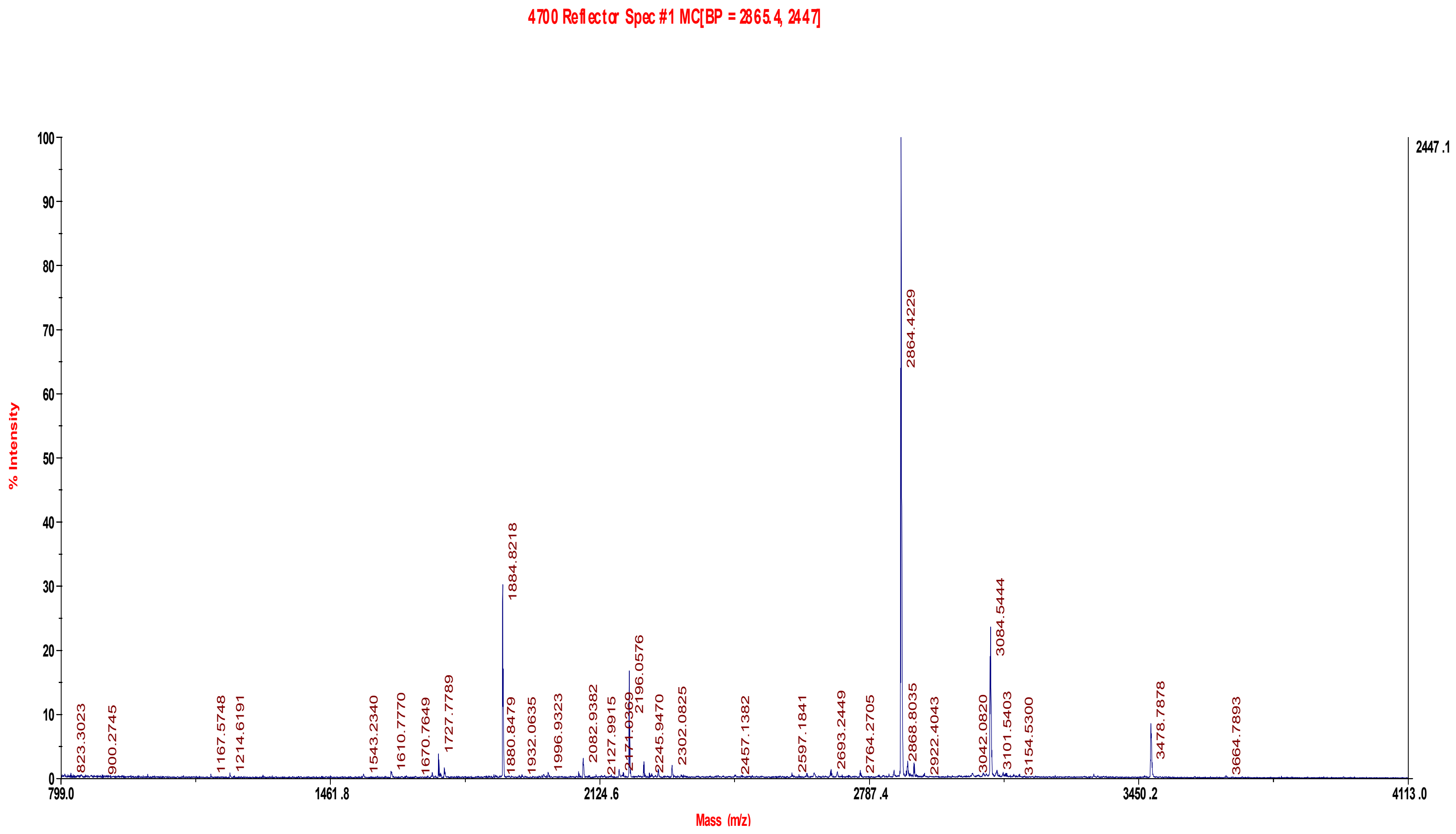

G. **Flagellar capping protein FliD**

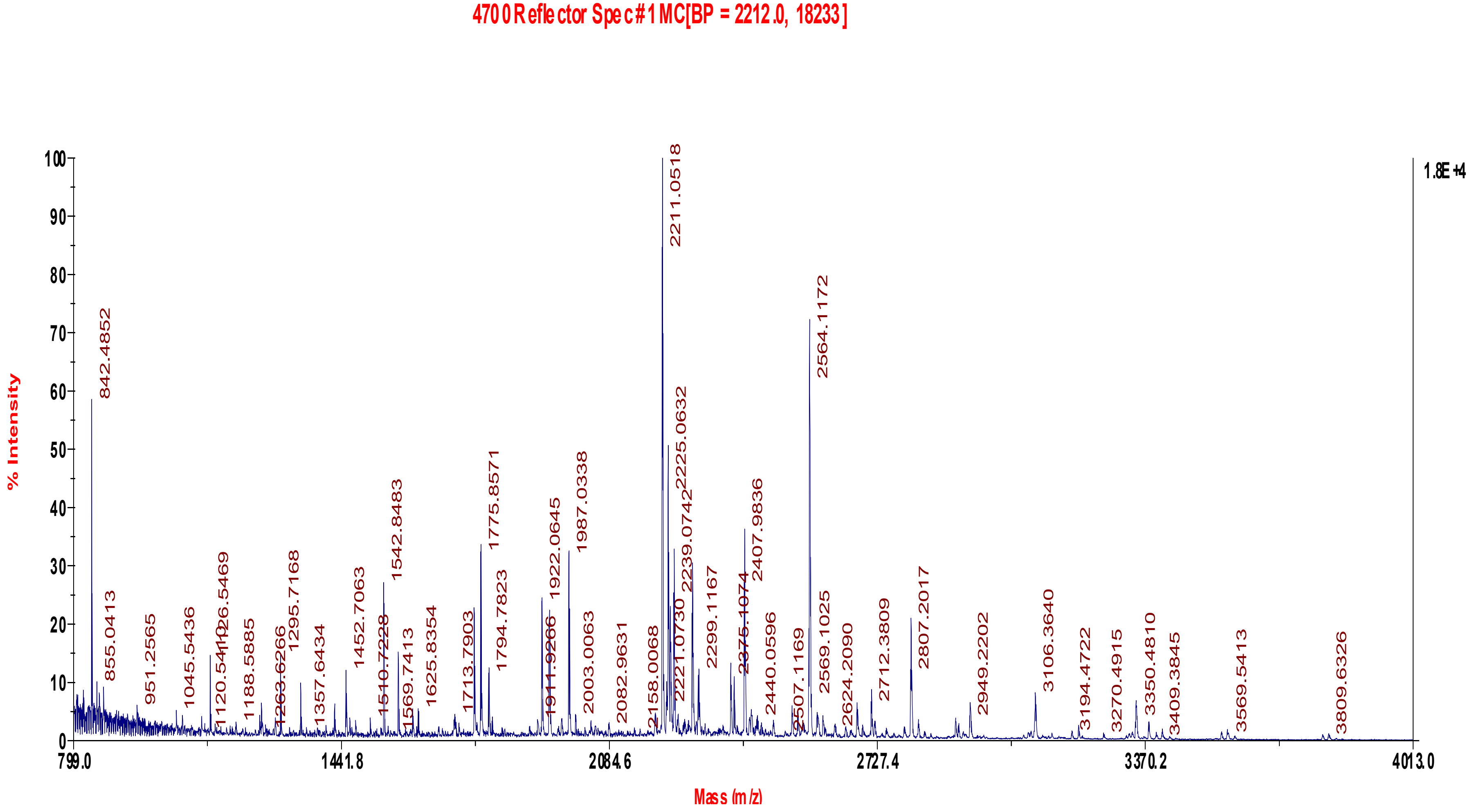

H. Elastase LasA

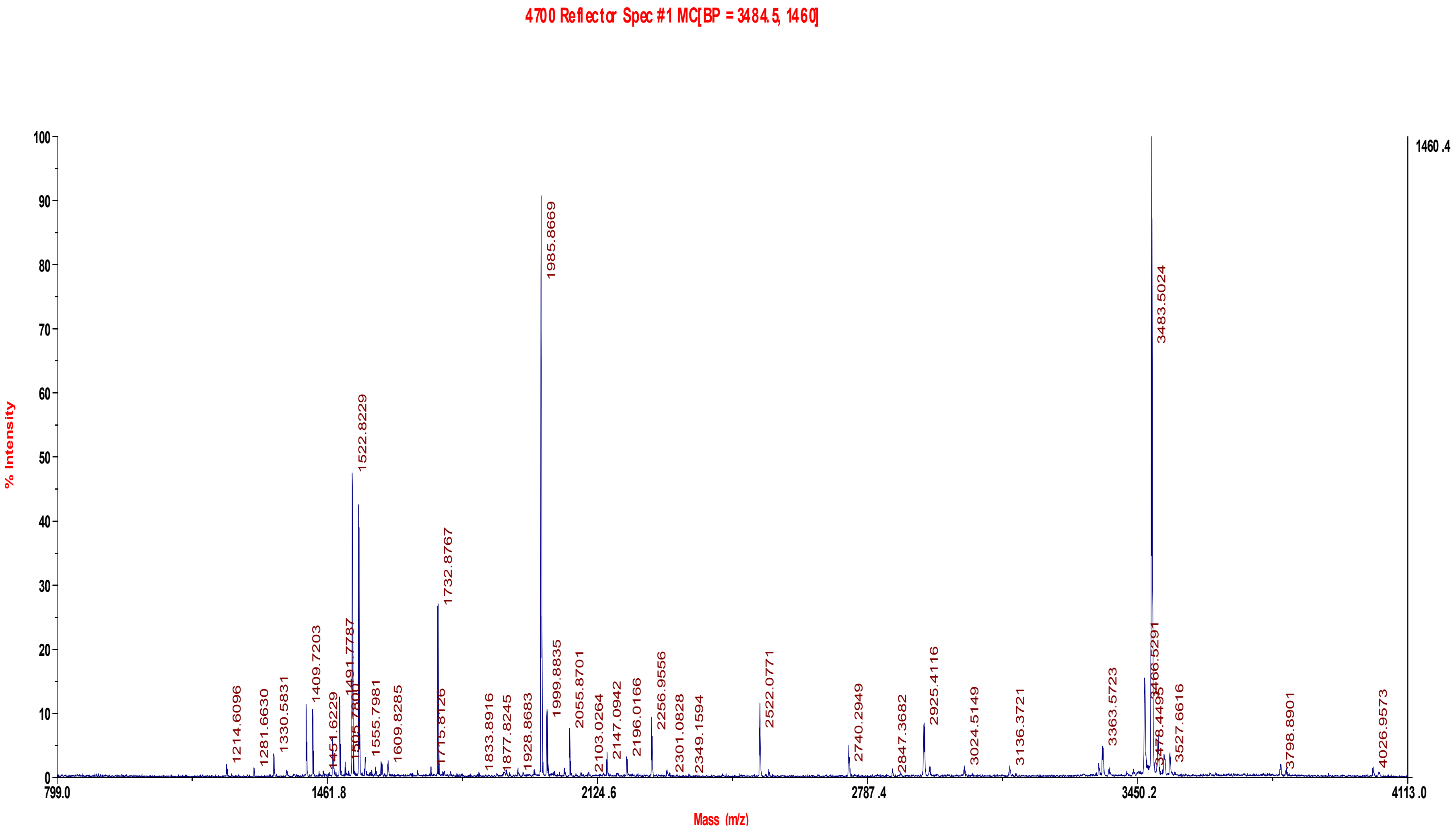

Supplement: S2 Fig — (PDF) [file pone.0123805.s002.pdf]
