# Supplementary material for: Global Regulator MorA Affects Virulence-Associated Protease Secretion in Pseudomonas aeruginosa PAO1
Source: PLoS One. 2015 Apr 20;10(4):e0123805. doi: 10.1371/journal.pone.0123805 (PMC4404142; doi:10.1371/journal.pone.0123805)
Supplement: S3 Fig — (PDF) [file pone.0123805.s003.pdf]

Global Regulator MorA affects Virulence-associated Protease Secretion in *Pseudomonas aeruginosa* PAO1

Supporting Information

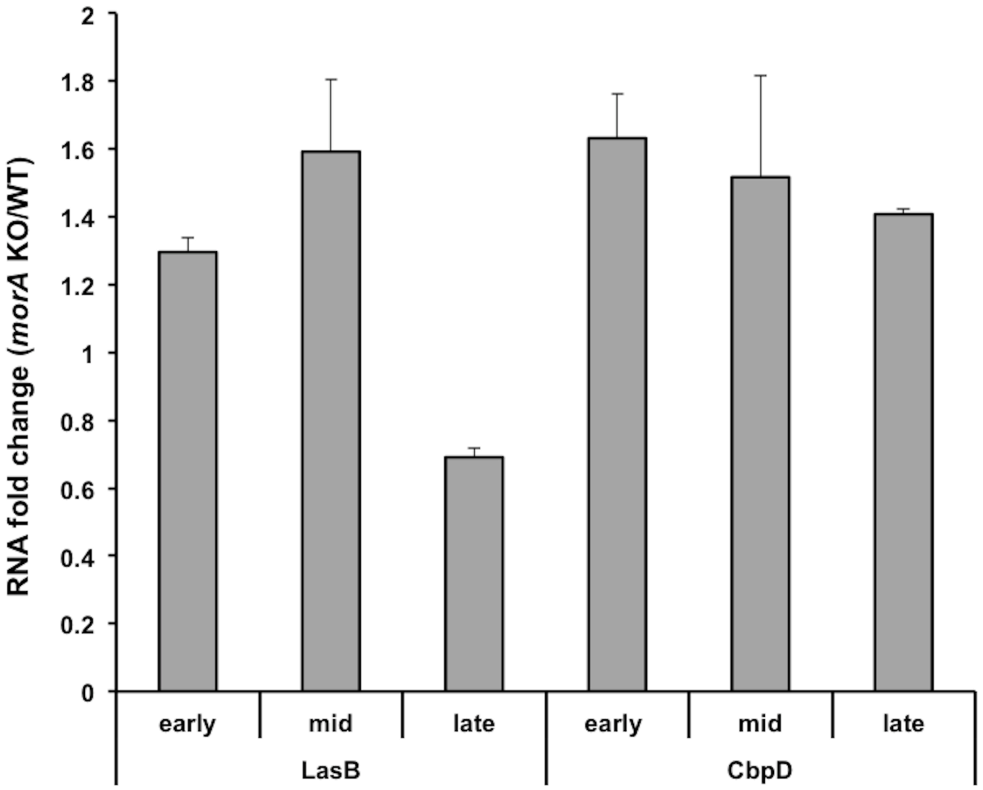

S3 Figure. RNA levels of major secreted proteases show no significant change due to MorA. Graph shows RNA transcript fold changes of *lasB* and *cbpD* at early-, mid- and late-log phases in *morA* KO with respect to WT by quantitative real-time PCR. All changes are less than two fold. The error bars represent mean +SE (n=3). Student's t-test, p-value < 0.05. Levels of *rpsL*, encoding 30S ribosomal protein was used as internal control.
