# Supplementary material for: Global Regulator MorA Affects Virulence-Associated Protease Secretion in Pseudomonas aeruginosa PAO1
Source: PLoS One. 2015 Apr 20;10(4):e0123805. doi: 10.1371/journal.pone.0123805 (PMC4404142; doi:10.1371/journal.pone.0123805)
Supplement: S1 Table — (PDF) [file pone.0123805.s004.pdf]

Global Regulator MorA affects Virulence-associated Protease Secretion in *Pseudomonas aeruginosa* PAO1

Supporting Information

S1 Table. List of primers used in this study

Primer	Primer sequence	
**Transcriptional analysis**		
qRT_RpsL_F	5' ACGTTGCGGG CAGTTCTG 3'	
qRT_RpsL_R	5' AGCCGCGTAA GCGTATCGT 3'	
LasB_qRT_Fwd	5′ CCAGGCCAAG AGCCTGAAG 3′	
LasB_qRT_Rev	5′ CGGATCACCA GTTCCACTTT G 3′	
CbpD_qRT_Fwd	5′ CCGGCAAGCA TGTGATCTAT AA 3′	
CbpD_qRT_Rev	5′ CGTCGATGCA GGCGTAGAA 3′	
**Cloning for recombinant protein expression**		
LasB_FwBamHI2	5′ CGCGGATCCA AGATCGGCAA GTACACCTAC GG 3'	
LasB_RvHindIII2	5' TCAGAAGCTT TTACAACGCG CTCGGGCAGG 3′	
**Site-directed mutagenesis of MorA enzymatic domains***		
E1060K_Up_Fwd	5′ CACTGGTTCG ACATCCGC 3′	2023-2040
E1060K_Up_Rev	5′ GCAGCAGGGC CTTGGCGCCG GTCAG 3′	3577-3553
E1060K_Dwn_Fwd	5′ CTGACCGGCG GCAAGGCCCT GC 3′	3553-3574
E1060K_Dwn_Rev	5′ TCAGCCCTCG TTGAACATG 3′	4248-4230
E1189K_Up_Fwd	5′ CACTGGTTCG ACATCCGC 3′	2023-2040
E1189K_Up_Rev	5′ CAGCAGGGTG AATTTGTCGC CGCCCAT 3′	3192-3166
E1189K_Dwn_Fwd	5′ ATGGGCGGCG ACAAATTCAC CCTGCTG3′	3166-3192
E1189K_Dwn_Rev	5′ TCAGCCCTCG TTGAACATG 3′	4248-4230

*The last column represents the primer position in *morA* gene
