# Supplementary material for: Global Regulator MorA Affects Virulence-Associated Protease Secretion in Pseudomonas aeruginosa PAO1
Source: PLoS One. 2015 Apr 20;10(4):e0123805. doi: 10.1371/journal.pone.0123805 (PMC4404142; doi:10.1371/journal.pone.0123805)
Supplement: S2 Table — (PDF) [file pone.0123805.s005.pdf]

Global Regulator MorA affects Virulence-associated Protease Secretion in *Pseudomonas aeruginosa* PAO1

Supporting Information

S2 Table. MALDI-ToF-ToF identification of *P. aeruginosa* secreted proteins affected by MorA. Peptide mass tolerance and fragment mass tolerance were 100ppm and 0.2 Da respectively. At the maximum, 1 missed cleavage for trypsin was allowed.

Band No.	No. of queries matches	Nominal Mass	Peptide sequences	Accession number	Genbank Identifier	Protein name/function	Sequence coverage
1	35	100615	1. MSLSTTAFPSLQGENMSRSPIPR<br>2. GLAYGTNVLTQLSGTNAAHAPLLKR<br>3. LLVLGNGASAASLSATVR<br>4. SVELGGAYGQDPALVQQIVDGSWR<br>5. AQNLFALPGTTSLR<br>6. LWLLWADAVR<br>7. AADTARFQETFVADAIVGYVR<br>8. FQETFVADAIVGYVR<br>9. QQSMPVSGSEETLTLTLPSAQGFTAIGR<br>10. LSIRIEDAGQASLAVGLNTQR<br>11. IEDAGQASLAVGLNTQR<br>12. LQANQSVALVSPYGGLLQLVYSGATPGQTVTVK<br>13. VTGAASQPFLDIQPGEDSSQAIADFIQALDADK<br>14. ADWLEIR<br>15. GWGESHELGHNLQVNR<br>16. SGEISNQIFPLHK<br>17. EFGQNLDDTR<br>18. NAYNLIVAGR<br>19. AEADPLAGVYK	PA0572	GI:15595769	Hypothetical protein	43%

			20. LWEDPGTYALNGER<br>21. MAFYTQWVHYWADLK<br>22. NDPLQGWDIWTLLYLHQR<br>23. DQRPTFALWGIR<br>24. TSAAAQAQVAAYGFAEQPAFFYANNR				
2	23	57824	1. SPLLVSTPLGLPR<br>2. LEDIASLNDGNR<br>3. AAATPGYQASVDYVK<br>4. VSVQPFPFTAYYPK<br>5. GPGSLSATVPQPVTYEWEKDFTYLSQTEAGDVTAK<br>6. DFTYLSQTEAGDVTAK<br>7. AENAAAAGAAGVIIFNQGNTDDRK<br>8. KTETYNVVAETR<br>9. FAWWGAEEAGLVGSTHYVQNLAPEEK<br>10. LFEAYFR<br>11. GQQSEGTEIDFR<br>12. SDYAEFFNSGIAFGGLFTGAEGLK<br>13. YGGTAGKAYDECYHSK	PA2939	GI:15598135	Proable aminopeptidase	39%
3	13	50402	1. SDAYTQVDNFLHAYAR<br>2. GGDELVNGHPSYTVDQAAEQILR<br>3. APGDSVLTLSYSFLTKPNDFFNTPWK<br>4. AYSVMSYWEEQNTGQDFK<br>5. GAYSSAPLLDDIAAIQK<br>6. TGDTVYGFNSNTER<br>7. LVFSVWDAGGNDTLDFSGFSQNQK<br>8. IDLSGLDAFVNGGLVLQYVDAFAGK	PA1249	GI:15596446	AprA| Alkaline metalloproteinase precursor	34%

4	16	42398	1. AAVAAGGTQALYDWNGVNQGNANGNHQAVVPDGQLCGAGK<br>2. SDWPSTAIAPDASGNFQFVYK<br>3. YFDFYITK<br>4. HVIYNVWQR<br>5. AQQDLPAGATVTLR<br>6. LFDAQGR<br>7. QWPLALAQK<br>8. VNQDSTLVNIGVLDAYGAVSPVASSQDNQVYVR<br>9. FQVDIELPVEGGGEQPGGDGK<br>10. VDFDYPQGLQQYDAGTVVR<br>11. GWDLYYAPGK	PA0852	GI:116048775	CbpD|Chitin binding protein	49%
5	20	33354	1. IGKYTYGSDYGPLIVNDR<br>2. YTYGSDYGPLIVNDR<br>3. FACPTNTYK<br>4. QVNGAYSPLNDAHFFGGVVFK<br>5. LYRDWFGTSPLTHK<br>6. DWFGTSPLTHK<br>7. GQSGGMNEAFSDMAGEAAEFYMR<br>8. GKNDFLIGYDIK<br>9. YMDQPSRDGR<br>10. YMDQPSR<br>11. SIDNASQYYNGIDVHHSSGVYNR<br>12. AFYLLANSPGWDTRK<br>13. AFYLLANSPGWDTR<br>14. AFEVFVDANR<br>15. YYWTATSNYNSGACGVIR<br>16. NYSAADVTR	PA3724	GI:15598919	LasB| Elastase	51%
6	9	48589	1. MVFTSSADGGSYICTGTLLNNGNSPK<br>2. TPPAGVFYQGWSATPIANGSLG	PA4175	GI:15599370	PrpL| PvdS regulated endoprotease;	31%

			HDIHHPR<br>3. YSQGNVSAVGVTYDGHTALTR<br>4. VDWPSAVVEGGSSGSGLLTVAGDGSYQLR<br>5. NDYFSDFSGVYSQISR			lysyl class	
7	19	49420	1. ASATQSAVAGTYQIQVNSLATSSK<br>2. IALQAIADPANAK<br>3. FNSGTLNISVGDTK<br>4. LPAITVDSSNNTLAGMR<br>5. DAINQAGKEAGVSATIITDNSGSR<br>6. VEVSDDGSGGNTSLSQLAFDPATAPK<br>7. AANGEITVDGLKR<br>8. SIASNSVSDVIDGVSFDVK<br>9. AVTEAGKPITLTVSR<br>10. LTTQFNLLSAMQDEMTK	PA1094	GI:15596291	FliD| Flagellar capping protein	38%
8	7	45721	1. AVGEDGLNAASAALLGLLREGAK<br>2. QGYSWQPNGAHSNTGSGYPYSSFDASYDWPR<br>3. WGSATYSVVAAHAGTVR<br>4. VTHPSGWATNYYHMDQIQVSNGQQVSADTK<br>5. YYFYNQSAGTTHCAFRPLYNPGLAL	PA1871	GI:15597068	LasA| Elastase	30%
